# Reversible Molecular Conformation Transitions of Smectic Liquid Crystals for Light/Bias-Gated Transistor Memory

**DOI:** 10.1021/acsami.3c16882

**Published:** 2024-02-01

**Authors:** Yi-Chieh Neu, Yi-Sa Lin, Yi-Hsun Weng, Wei-Cheng Chen, Cheng-Liang Liu, Bi-Hsuan Lin, Yan-Cheng Lin, Wen-Chang Chen

**Affiliations:** † Department of Chemical Engineering, National Taiwan University, Taipei 10617, Taiwan; ‡ Department of Materials Science and Engineering, National Taiwan University, Taipei 10617, Taiwan; § Advanced Research Center for Green Materials Science and Technology, National Taiwan University, Taipei 10617, Taiwan; ∥ National Synchrotron Radiation Research Center, Hsinchu 30076, Taiwan; ⊥ Department of Chemical Engineering, National Cheng Kung University, Tainan 70101, Taiwan

**Keywords:** smectic liquid crystal, rod-like molecules, homeotropic alignment, photomemory, field-effect transistors

## Abstract

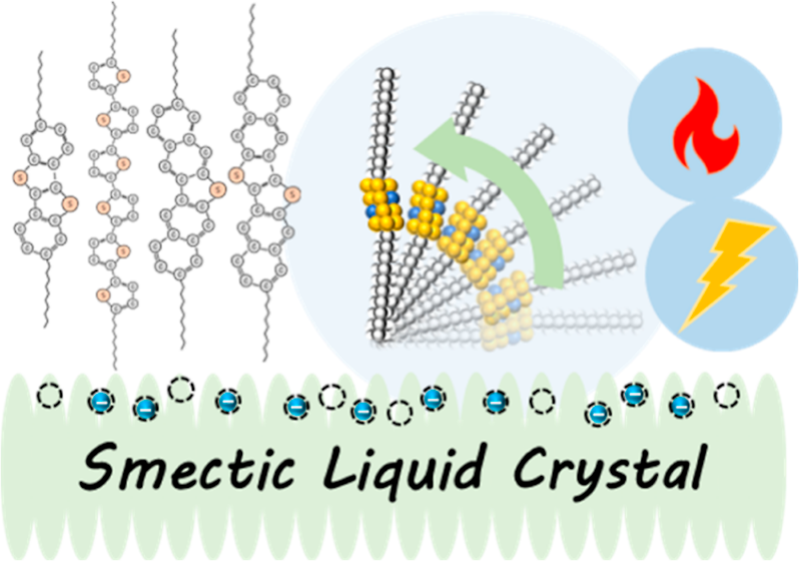

In recent years, organic photonic field-effect transistors have made remarkable progress with the rapid development of conjugated polycrystalline materials. Liquid crystals, with their smooth surface, defined layer thickness, and crystalline structures, are commonly used for these advantages. In this work, a series of smectic liquid crystalline molecules, 2,9-didecyl-dinaphtho-thienothiophene (C10-DNTT), 2,7-didecyl-benzothieno-benzothiopene (C10-BTBT), 3,9-didecyl-dinaphtho-thiophene (C10-DNT), and didecyl-sexithiophene (C10-6T), have been used in photonic transistor memory, functioning as both hole-transport channels and electron traps to investigate systematically the reasons and mechanisms behind the memory behavior of smectic liquid crystals. After thermal annealing, C10-BTBT and C10-6T/C10-DNTT are homeotropically aligned from the smectic A and smectic X phases, respectively. The 3D-ordered structure of these smectic-aligned crystals contributed to efficient photowriting and electrical erasing processes. Among them, the device performance of C10-BTBT was particularly significant, with a memory window of 21 V. The memory ratio could reach 1.5 × 10^6^ and maintain a memory ratio of over 3 orders after 10,000 s, contributing to its smectic A structure. Through the research, we confirmed the memory and light/bias-gated behaviors of these smectic liquid crystalline molecules, attributing them to reversible molecular conformation transitions and the inherent structural inhomogeneity inside the polycrystalline channel layer.

## Introduction

In the past few years, the performance of organic field-effect transistors (OFETs) has been achieved with significant advancements. The use of liquid crystal materials is increasingly substantial since liquid crystals possess the characteristics required for electronic devices, including a smooth surface morphology, a well-defined layer thickness, tunable thermotropic alignment, and crystalline structures.^1−3^ Additionally, it can enhance their crystallinity after postthermal annealing at the smectic phase transition temperature region, which provides high mobility in the FETs. Thermotropic liquid crystals can be broadly categorized into three primary types: calamitic (rod-like molecules), discotic (disk-like molecules), and cholesteric (chiral rod-like molecules).^4−6^ Within the calamitic type, there are further divisions into nematic, smectic, and cholesteric phases. In a nematic mesophase, liquid crystal molecules exhibit a one-dimensional regular alignment in space. The long axes of all molecules choose a specific direction as the central axis and align parallel to each other. The cholesteric mesophase is formed by stacking multiple layers of nematic mesophase, where the long axes of molecules in each layer gradually differ at a certain angle, resulting in an overall helical structure.^7−9^

Smectic liquid crystals are a type of thermotropic liquid crystal that exhibit a layered structure with a long-range order in the direction perpendicular to the layers.^10,11^ In terms of spatial ordering, they show an additional one-dimensional regularity compared to that of nematic liquid crystals, making them a layered structure. They can also be further classified into more than 11 different types of smectic liquid crystals, from smectic A (SmA) to smectic K (SmK), depending on the arrangement of molecules and interlayer structures.^12^ For example, in the liquid-like SmA and smectic C (SmC) phases, the molecules in the former align their long axes along the normal direction within each layer, while in the latter, the molecules exhibit a tilt at a specific angle along the smectic plane.^13^ In the solid-like smectic E (SmE) and smectic G (SmG) phases, the former presents a herringbone-type stacking,^13^ while the latter is influenced by the molecules above and below,^14^ resulting in a certain three-dimensional (3D) order.

Smectic liquid crystals are a class of liquid crystals that offer several advantages across various technological and optoelectronic/display applications; they are characterized by their highly ordered molecular arrangements, and this high degree of molecular order transforms into exceptional optical and electronic properties. In addition, smectic liquid crystals also exhibit impressive electrical properties, especially certain phases with ferroelectric characteristics. These phases allow for fast molecular realignment under the influence of an electric field, and this feature is favorable for phase-change and ferroelectric memory applications. Smectic liquid crystals are renowned for their stability, which makes them able to operate effectively and maintain long-term reliability. This durability makes smectic liquid crystals ideal for use in industry and research. Not only in these traditional electronic products, but smectic liquid crystals have also found extensive applications in electronic devices, such as field-effect transistors,^15−17^ solar cells,^18−20^ and nonvolatile memory devices.^21,22^ Transistor memory has evolved from field-effect transistors by adding an additional memory layer; they possess different mechanisms, such as floating-gate, charge-trapping, and ferroelectric dielectrics.^23^ Recently, photo memory has shown many advantages because of the perspective of developing light-assisted multibit data storage in a single memory cell with a high memory access speed. One of the promising avenues of development is photonic transistor memory, which has the potential to achieve high-speed communication, low energy cost,^24^ and multilevel storage capabilities.^25,26^ The design of organic molecules has garnered widespread attention in the study of memory effects. Small molecules offer numerous advantages,^27−30^ such as well-defined molecular structures and ordered intermolecular arrangements, which make them indispensable in this regard.

Smectic liquid crystals possess highly ordered stacking and orientation, leading to high mobility in terms of transistors. While smectic liquid crystals hold great promise in various aspects, they have certain drawbacks in OFET or solar cell devices. Specifically, smectic-type liquid crystals often require a single or two alkyl side chains to achieve high mobility. However, this comes at the expense of lower thermal stability, which is not conducive to their applications. This light/bias instability/hysteresis induces broad research interest in utilizing this feature in memory device applications. Recent studies have noted that organic crystalline molecules inherently possess a molecular layered structure, which may result in morphological inhomogeneity and potentially induce memory behavior.^31,32^ However, the reasons and mechanisms behind the memory behavior of these molecules, especially those for smectic liquid crystals, have not been systematically investigated. Zheng et al. conducted a study on temperature control during the deposition of organic molecules through vapor deposition. They aimed to compare whether there are differences related to nanosprout. They discovered that the source of memory behavior does not stem from dielectric or channel impurities but instead results from structure inhomogeneity.^33,34^ Consequently, this structure inhomogeneity gives rise to memory behavior, leading to hysteresis phenomena in both the optical response and electric fields. Hu et al. found that when organic molecules form phase-changeable organic semiconductors, they exhibit hysteresis phenomena in response to heat and electric fields, making them suitable for use in phase-change transistor memory. This light/bias hysteresis is due to reversible molecular conformation transitions.^35^ Therefore, it is also possible that it originates from these two issues in the memory behavior of liquid crystals.

To gain a deeper understanding of the memory behavior of liquid crystal molecules, we have applied a series of rod-like molecules, 2,9-didecyldinaphtho[2,3-*b*:2′,3′-*f*]thieno[3,2-*b*]thiophene (C10-DNTT), 2,7-didecyl[1]benzothieno[3,2-*b*][1]benzothiopene (C10-BTBT), 3,9-didecyldinaphtho[2,3-*b*:2′,3′-*d*]thiophene (C10-DNT), and 5,5‴″-didecyl-2,2’:5′,2″:5′,2‴:5‴,2‴′:5″″,2‴″-sexithiophene (C10-6T). The side-chain length of these rod-like molecules is carefully selected to control the phase transition region of smectic liquid crystals. In addition, these four materials are all common smectic liquid crystal materials with a close molecular size and comparable carrier mobilities. However, they are typically employed in transistors rather than in memory devices.^36−39^ The resultant thermal properties were characterized using differential scanning calorimetry (DSC); the optical properties were characterized using time-resolved photoluminescence (TRPL); the surface morphologies were characterized using polarized optical microscopy (POM), scanning electron microscopy (SEM), atomic force microscopy (AFM), and grazing incidence X-ray diffraction (GIXD). These molecules were applied to devices, and their hysteresis phenomena were investigated under light and electric fields. The results indicate that memory phenomena are observed in both as-deposited and molecular flat structures that are homeotropically aligned by annealing in the temperature ranges of liquid crystalline mesophases. Therefore, our findings feature the fact that reversible molecular conformation transitions and inherent structure inhomogeneity have the potential for developing nonvolatile memory with a combined single-layered structure of semiconducting/memory layers.

## Experimental Section

### Materials

2,9-Didecyldinaphtho[2,3-*b*:2′,3′-*f*]thieno[3,2-*b*]thiophene (C10-DNTT), 2,7-didecyl[1]benzothieno[3,2-*b*][1]benzothiopene (C10-BTBT), 3,9-didecyldinaphtho[2,3-*b*:2′,3′-*d*]thiophene (C10-DNT), and 5,5‴″-didecyl-2,2′:5′,2″:5″,2‴:5‴,2‴′:5‴′,2‴″-sexithiophene (C10-6T) were purchased from Luminescence Technology Corp. (LUMTEC, Hsinchu, Taiwan).

### Characterization

The thermal properties were characterized by using TA Instruments TGA55 and DSC25 systems for thermogravimetric analysis (TGA) and DSC analyses. For the optical properties, UV–vis absorption spectroscopy was measured by using a Hitachi U-4100 spectrophotometer. PL emission spectroscopy was measured using a HORIBA Jobin Yvon Fluorolog-3 spectrofluorometer. TRPL spectroscopy was conducted at a wavelength of 375 nm and collected using a fiber coupled with a Hamamatsu C10910 streak camera and an M10913 slow single-sweep unit with an instrument response function of 82 ps at the National Synchrotron Radiation Research Center (NSRRC), Taiwan. The 1D decaying profiles were fitted by using an exponential decay function to derive the exciton lifetime (τ), as shown in the following eq 1
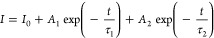
1

Cyclic voltammetry (CV) was performed using a CHI 6273E electrochemical analyzer in which Ag/AgCl and Pt rods were used as a reference and a counter electrode, respectively. The morphology of polycrystalline films of the rod-like molecules was investigated by an OLYMPUS BX51 optical microscope with a U-POT polarizer, JSM-7600F Schottky field-emission SEM, and Bruker Innova AFM under tapping mode. GIXD analysis of the rod-like molecule films was collected on beamline TLS BL13A1 in NSRRC, Taiwan. The X-ray beam with a wavelength of 1.03 Å was used, and the incident angle was set as 0.12°.

### Fabrication and Characterization of the Photonic Transistor Memory Devices

A photonic transistor memory device was fabricated based on a bottom-gate/top-contact configuration on a highly n-doped Si substrate with a 100 nm thick SiO_2_ layer. The wafer surface was initially cleaned with isopropyl alcohol by an ultrasonic cleaner to remove contaminants. Then, the rod-like molecules were deposited onto the substrate through thermal evaporation, forming a 50 nm thick polycrystalline film. The deposition rate was 0.2–0.3 Å s^–1^ under a 10^–7^ Torr vacuum. Next, they were annealed individually at 100 or 150 °C, depending on their phase transition regions, under a vacuum for an hour. Finally, Au was deposited onto the above samples by thermal evaporation through a regular shadow mask, forming 70 nm thick gold electrodes. Note that the channel length (*L*) and width (*W*) were 1000 and 50 μm, respectively. The photonic transistor memory device was characterized using a Keithley 4200-SCS semiconductor parameter analyzer in a N_2_-filled glovebox. The measurements were conducted in a dark environment to reduce external disturbance. All illumination was performed using LED systems equipped with 310 and 365 nm light. For the transfer characteristics, *V*_d_ was set as −50 V, and the gate voltage was swept from *V*_g_ = 30 to −50 V. Photowriting was conducted at *V*_d_ = −50 V and *V*_g_ = 0 V. Electrical erasing was conducted by applying *V*_g_ (−60 V, 5 s) at *V*_d_ = 0 V.

## Results and Discussion

### Thermal Characterizations of the Rod-like Molecules

**Figure 1:**
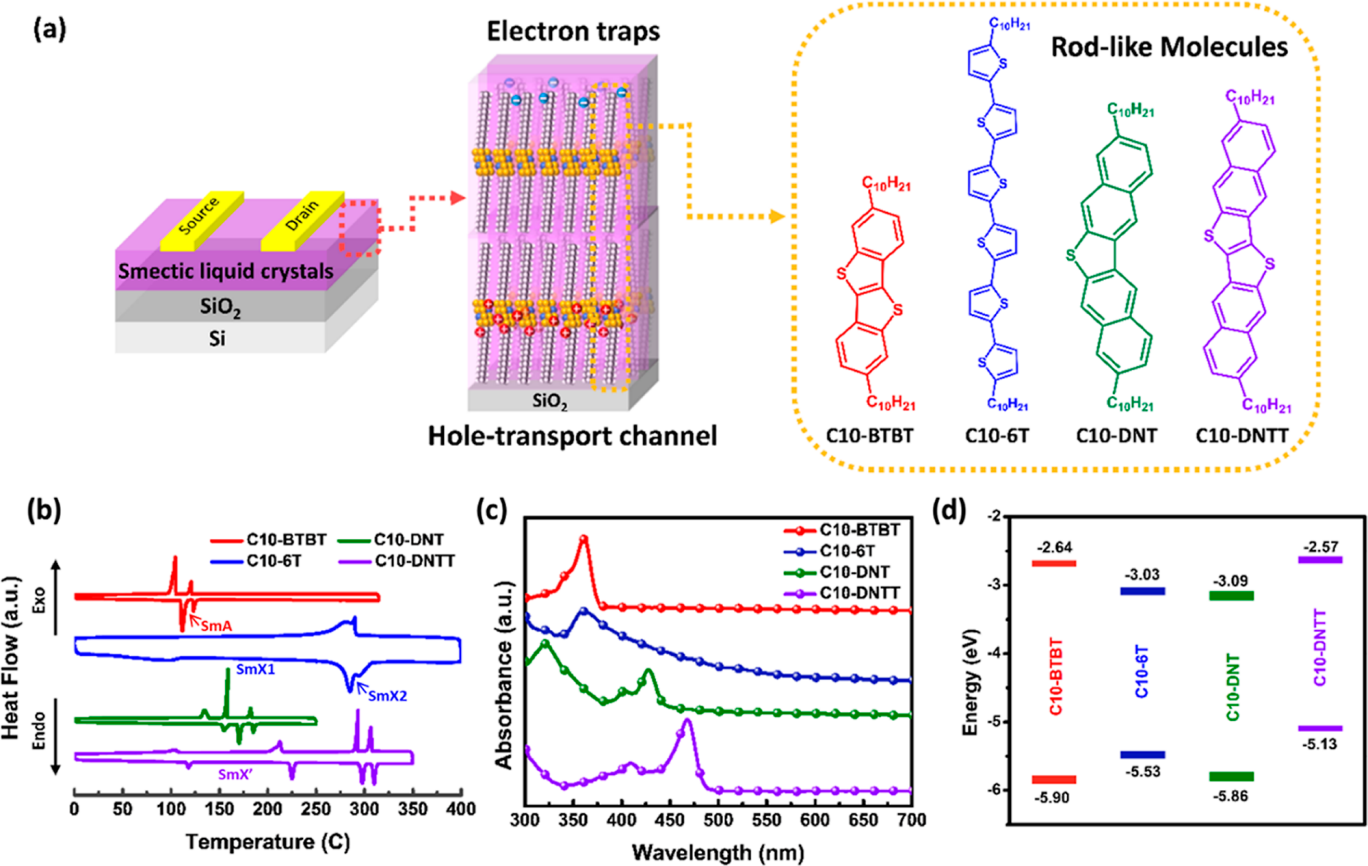
(a) Schematic diagram of the photonic transistor memory comprising rod-like molecules and their chemical structures. (b) DSC profiles, (c) UV–vis absorption spectra, and (d) energy level diagram of the constituent rod-like molecules.

In order to understand the impact of liquid crystal molecules on the conformation and device performance of photonic transistors under external stress, we designed four rod-like molecules, C10-BTBT, C10-6T, C10-DNT, and C10-DNTT, applied in OFET devices. Figure 1a shows the device structure diagram and the chemical structures of these rod-like molecules. All of these molecules possess a typical p-type semiconductor core. In this layered structure, the lower layer may serve as the hole-transport channel, while structures away from the channel potentially exhibit electron-trapping effects. This interplay will be further discussed in the device Characterization section.

To understand the phase transition behavior of these rod-like molecules, we conducted thermal analyses of TGA and DSC. TGA profiles are listed in Figure S1 (Supporting Information), and DSC profiles are shown in Figure 1b. The rod-like molecules exhibit high thermal stability with thermal decomposition over 300 °C. In the heating ramp of DSC, C10-BTBT exhibits two distinct peaks at 112 and 123 °C, representing the smectic phase transition points and the melting point, respectively. C10-6T exhibits one broad and two distinct peaks at 108 and (280, 290) °C.^40^ The first two peaks represent the smectic phase transition points, while the last corresponds to the melting point. Note that SmX1 and SmX2 are different smectic phases. C10-DNT exhibits three distinct peaks at 155, 170, and 185 °C.^41^ The first peak represents the smectic phase transition point, and the last corresponds to the melting point. C10-DNTT exhibits four distinct peaks at 118, 224, 298, and 310 °C.^42,43^ The first peak represents the smectic phase transition point, while the last corresponds to the melting point. The findings align with the reported observations in the literature.^40−44^

To gain insight into the phase transitions in their thin film states, POM and conoscopic images in Figures S2 and S3 (Supporting Information) indicate that C10-BTBT enters the SmA phase at 112 °C,^44^ followed by reaching the melting point beyond 125 °C; the observable focal conics affirm its SmA phase. The transformation could also be identified in C10-DNT at 160, 170, and 180 °C; C10-6T at 280 °C; C10-DNTT at 150, 250, and 300 °C. Upon cooling to room temperature, they crystallized into polycrystalline films. By using a built-in Bertrand lens accessory to obtain the conoscopic image, however, the surface of a drop-casted film (around a hundred micrometers in thickness) is not flat enough to facilitate the light interference to derive an obvious optical axis pattern. To objectively compare the solid-state stacking of the rod-like molecule films, we compared their as-deposited thin films with those annealed at 100 °C for C10-BTBT and C10-6T and 150 °C for C10-DNT and C10-DNTT to enhance their homotropic alignment. Accordingly, C10-BTBT, C10-6T, and C10-DNTT were homeotropically aligned from the SmA, SmX, and SmX′ phases, respectively.^36,39,40^ Note that SmX and SmX′ are not the same smectic phases.

### Optical Characterizations of the Polycrystalline Films

With Figure 1c presenting their UV–vis absorption spectra, C10-BTBT, C10-DNT, and C10-DNTT show characteristic absorption at 330–380, 300–440, and 400–480 nm, respectively, and C10-6T shows a broader absorption band spanning the range of 200–500 nm. The absorption spectra of these polycrystalline films at different annealing temperatures are presented in Figure S4 (Supporting Information). As can be seen, the polycrystalline films exhibit similar optical absorption profiles after thermal annealing. C10-DNT presents a more blue-shifted absorption band than C10-BTBT and C10-DNTT because of its shorter conjugation length and the inherent weaker molecular aggregation and stacking attributed to the bent conjugated core. Figure S5 (Supporting Information) displays their PL emission spectra, with emission peak wavelengths falling within the range of 360–450, 400–570, 430–550, and 460–560 nm for C10-BTBT, C10-6T, C10-DNT, and C10-DNTT, respectively. Photoluminescence quantum yield (PLQY) was measured to acquire the photon conversion efficiency, and the values are 0.26, 0.23, 0.13, and 0.05% for C10-DNTT, C10-BTBT, C10-DNT, and C10-6T, respectively. The optical absorption and emissions of these rod-like molecules align with their conjugated core of dinaphtho-thienothiophene (DNTT), benzothieno-benzothiopene (BTBT), dinaphtho-thiophene (DNT), and sexithiophene (6T) for C10-DNTT, C10-BTBT, C10-DNT, and C10-6T, respectively. Next, to understand their energy levels, CV was utilized to calculate their highest occupied molecular orbital (HOMO) levels based on their oxidative onset (Figure S6, Supporting Information). Additionally, the results indicate that C10-DNT, with a phase transition temperature of >150 °C, has the same oxidative potential for both annealed and as-cast samples. In contrast, C10-6T, C10-DNTT, and C10-BTBT have positively shifted oxidation onset. This disparity may be related to their lower phase transition temperatures below 100–150 °C. Therefore, the homeotropic alignment of LC molecules may impose an overpotential on the CV measurement. The thin films in the as-deposited state were applied for energy level calculations. The lowest unoccupied molecular orbital (LUMO) levels were determined by combining the optical bandgap (*E*_g_) and HOMO levels: LUMO = HOMO + *E*_g_. The *E*_g_ was obtained from the UV–vis absorption onset. The calculated values are listed in Figure 1d. Their HOMO/LUMO levels are (−5.90, −2.64) eV, (−5.53, −3.03) eV, (−5.86, −3.09) eV, and (−5.13, −2.57) eV for C10-BTBT, C10-6T, C10-DNT, and C10-DNTT, respectively. The energy levels of these rod-like molecules align with their conjugated cores.^39,43,46^

**Figure 2:**
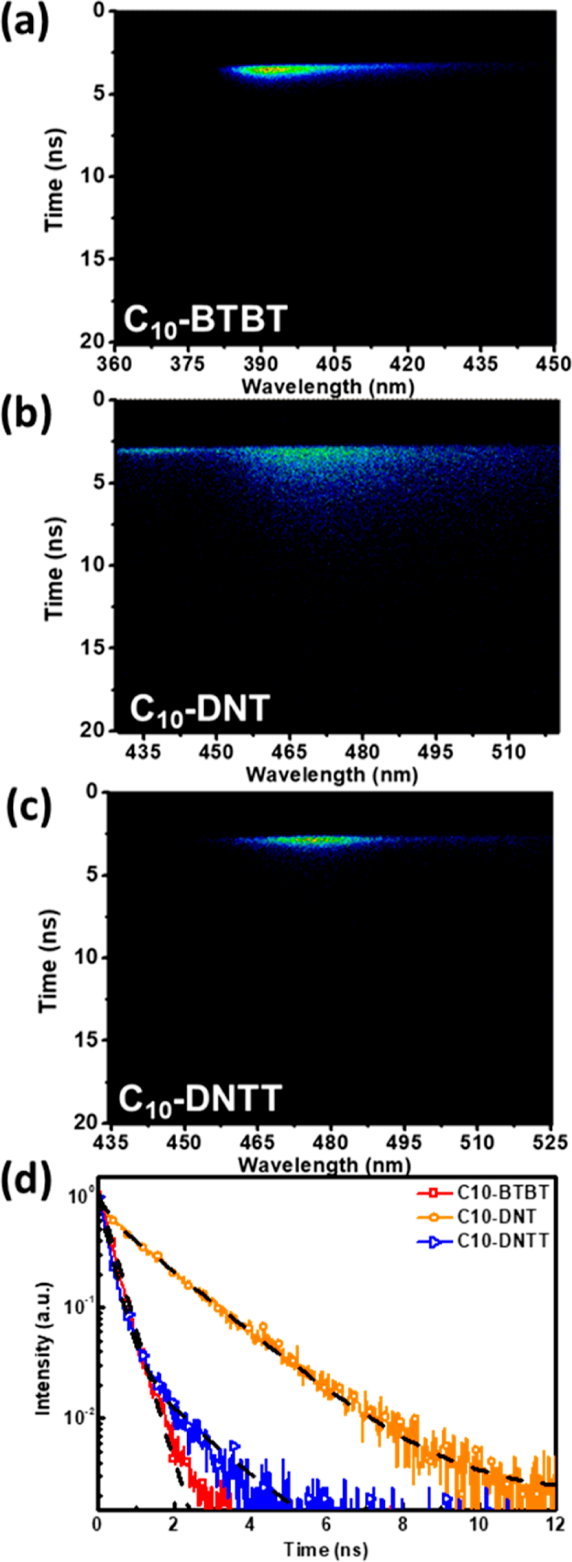
2D TRPL patterns of (a) C10-BTBT, (b) C10-DNT, and (c) C10-DNTT. (d) 1D TRPL decaying profiles of the rod-like molecule films.

After discussion of their steady-state optical properties, to understand their transient photoresponse behavior in devices, TRPL characterization was applied to study their photodynamic properties.^45^Figure 2a–c presents the 2-D TRPL data for the liquid crystal films except for C10-6T, where PL emission is too weak to detect. Figure 2d shows their 1D decay profile extracted from the 2D TRPL patterns. The fitting parameters are summarized in Table S1 (Supporting Information). The TRPL emission bands correspond to the PL emission spectra tracked in Figure S5 (Supporting Information). The average lifetime (τ_avg_) is calculated by τ_avg_ = *A*_1_τ_1_^2^/(*A*_1_τ_1_ + *A*_2_τ_2_) + *A*_2_τ_2_^2^/(*A*_1_τ_1_ + *A*_2_τ_2_). As can be seen, C10-DNT exhibits longer exciton lifetimes, while C10-BTBT and C10-DNTT have shorter lifetimes, measuring 1.51, 0.35, and 0.50 ns, respectively. Molecules with tightly stacked conjugated structures exhibit shorter lifetimes, while those with poorer conjugation/stacking exhibit longer lifetimes. C10-BTBT and C10-DNTT possess more rigid conjugated structures, leading to stronger aggregation and easier exciton quenching. In contrast, C10-DNT has weaker conjugation, resulting in poorer aggregation stacking and longer lifetimes. This disparity in molecular aggregation/packing aligns with the more blue-shifted optical absorption of C10-DNT.

### Morphological Characterizations of the Polycrystalline Films

**Figure 3:**
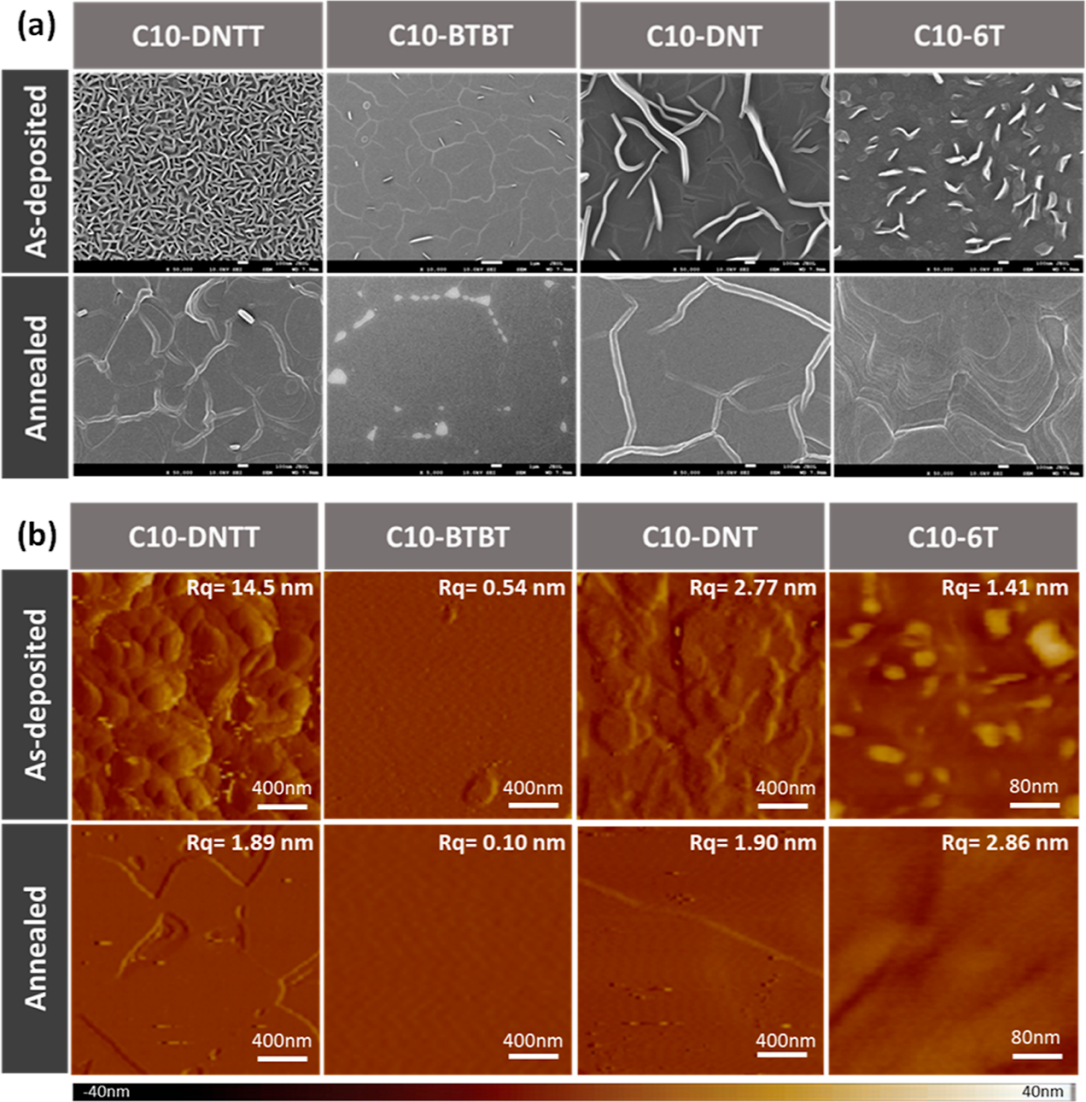
(a) SEM images and (b) AFM topographies of the as-deposited and annealed thin films of the rod-like molecule films. The polycrystalline films were 150 °C thermally annealed C10-DNTT, 100 °C thermally annealed C10-BTBT, 150 °C thermally annealed C10-DNT, and 100 °C thermally annealed C10-6T to promote the solid-state stacking by homeotropic alignment.

After understanding the optical properties of these rod-like molecules, their morphology was subsequently investigated. SEM images (Figure 3a) reveal a molecular flat structure formed immediately after vacuum deposition,^46^ and the rod-like molecules exhibited appropriate homeotropic alignment behaviors. Upon annealing, inhomogeneous sprout structures of the surface further developed into a flat configuration. The cooling rate of the annealed films in Figure 3a is at approximately 5 °C min^–1^. By slowing the cooling rate to 1 °C min^–1^, numerous sprouts formed on the surface, as shown in Figure S7 (Supporting Information). In contrast, by quenching the sample to 20 °C within 1 s, a flatter pattern could be observed. These observations may indicate that an inhomogeneous sprout structure develops into a flat configuration during the annealing process.

To characterize the surface conditions, we measured the morphologies of the prepared samples. AFM topographies (Figure 3b) showed structures similar to those in SEM images but provided a more in-depth exploration of height variations. The roughness values for the as-deposited and annealed C10-DNTT, C10-BTBT, C10-DNT, and C10-6T are (14.5, 1.89) nm, (0.54, 0.10) nm, (2.77, 1.90) nm, and (1.41, 2.86) nm, respectively. It can be observed that the roughness of C10-DNTT, C10-BTBT, and C10-DNT decreased after annealing. However, for C10-6T, the roughness increased after annealing compared to that in the as-deposited state. We further measured the surfaces of molecular flat platforms (Figure S8), and the lengths of the LC molecules are 4.2, 3.9, 4.0, and 6.2 nm for C10-DNTT, C10-BTBT, C10-DNT, and C10-6T, respectively. We can observe that the average roughness values of the surfaces are all smaller than one molecular length, yielding reliable flatness.

The morphological transitions of these polycrystalline films can be evaluated by variations in surface energy. Figure S9 shows that the surface energies of C10-DNTT, C10-DNT, and C10-6T increased after thermal annealing because the nanosprouts formed after vacuum deposition were hydrophobic. As a result of the relatively hydrophilic nature of the molecular flat surface, it can be deduced that after annealing, the rod-like molecules were oriented toward the surface, exhibiting an end-on conformation. In the case of C10-BTBT, it already exhibited a molecular flat surface in the as-deposited state, so annealing does not cause an increase in surface energy. In contrast, C10-BTBT’s surface energy decreased after thermal annealing, implying better flatness.

**Figure 4:**
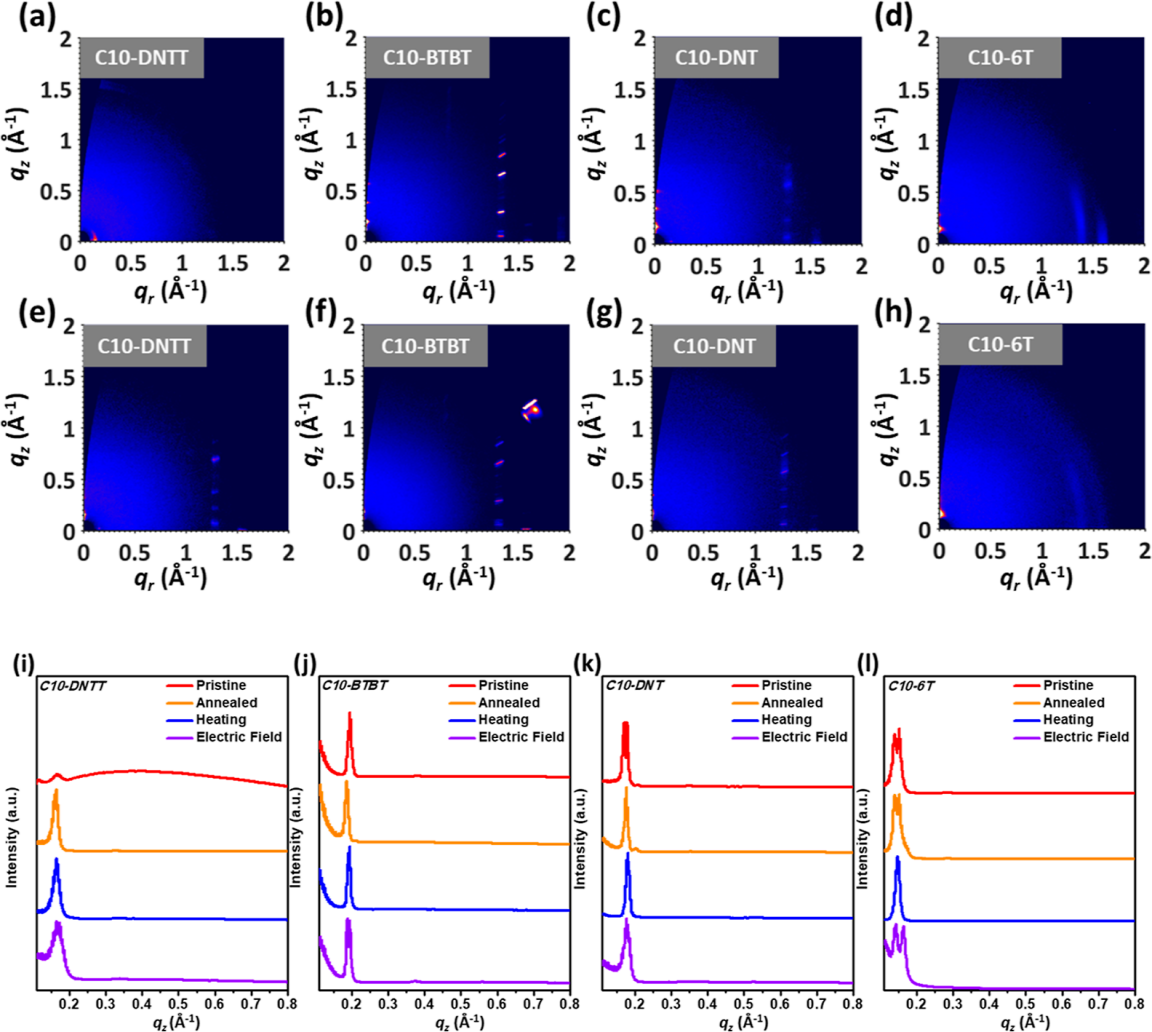
2D GIXD patterns of the (a–d) as-deposited and (e–h) thermally annealed rod-like molecule films. 1D line-cutting profiles of (i) C10-DNTT, (j) C10-BTBT, (k) C10-DNT, and (l) C10-6T along the out-of-plane direction. Note that the measurements were based on the as-deposited (pristine) or thermally annealed rod-like molecule films and the thermally annealed thin films under different treatments, including heating at 100 °C for C10-BTBT and C10-6T and 150 °C for C10-DNTT and C10-DNT (heating) or applying a vertical bias of 2 V (electric field).

The surface morphology observations can only point out the evolutions during vacuum deposition and homeotropic alignment during thermal annealing. GIXD analysis can gain further insight into the variations in crystallographic parameters. As shown in Figure 4a, the 2D GIXD patterns of the as-deposited and thermally annealed thin films were performed to verify how the annealing process affected the orientation of these rod-like molecules. The corresponding 1D line-cutting profiles along the out-of-plane direction were extracted and are presented in Figure 4b–e. In addition, in situ external stimuli, including thermal and electric fields, were applied during the measurements. The applied heat was set at the same temperature as the annealing temperatures, that is, 100 °C for C10-BTBT and C10-6T and 150 °C for C10-DNTT and C10-DNT. In addition, an out-of-plane electric field was conducted, with a voltage of 2 V and a fixed gap between two electrodes, during the entire measurement process, and the device diagram and fabrication for this measurement are presented in Figure S10 (Supporting Information). As the crystallographic parameters show in Table S3 (Supporting Information), the interlayer distances (*d*_001_) of (pristine, annealed, heating, bias) films were (3.8, 3.8, 3.8, 3.8) nm, (3.2, 3.4, 3.2, 3.3) nm, (3.6, 3.6, 3.5, 3.6) nm, and (4.1, 4.1, 4.3, 4.4) nm for C10-DNTT, C10-BTBT, C10-DNT, and C10-6T, respectively. Additionally, the crystallite sizes (*L*_c_) were calculated by the Scherrer equation, *L*_c_ = 0.9 × 2π/fwhm, where fwhm is the full-width at half-maximum of the (001) peak in the out-of-plane direction. The corresponding *L*_c_ were (19, 33, 28, 23), (47, 51, 57, 40), (33, 51, 47, 30), and (20, 21, 8, 6) nm under pristine, annealed, heating, bias conditions for C10-DNTT, C10-BTBT, C10-DNT, and C10-6T, respectively. Most polycrystalline films show a similar or increased spacing after thermal annealing. The external stimuli further altered the crystallographic parameters, which align with the characteristic behavior where rod-like molecules exhibit better stacking. C10-DNTT showed an unchanged *d*_001_ under different conditions owing to its highly rigid rod of DNTT. Notably, the *L*_c_ of these polycrystalline films decreased significantly when the bias was applied; in comparison, heating at 100–150 °C is not that capable of inducing the conformation changes. The results indicate that on applying external stimuli of heat and bias, the smectic liquid crystal molecules undergo molecular conformation changes, altering their alignments. Along the in-plane direction, the 1D line-cutting profiles are presented in Figure S11. After annealing, distinct peaks of (010) appear in C10-DNTT, C10-BTBT, and C10-DNT, indicating their π–π stacking. The spacings are 0.48, 0.48, and 0.47 nm for C10-DNTT, C10-BTBT, and C10-DNT, respectively, indicating that the intermolecular distances are similar. In addition, C10-6T presents a denser π–π stacking distance of 0.45 nm. For the azimuthal analysis of GIXD patterns, Figure S12 (Supporting Information) exhibits the geometrically corrected and normalized pole figures of the liquid crystal films. As can be seen, the films presented a predominantly end-on orientation and better crystallinity after thermal annealing.

### Photonic Transistor Memory Characterizations of the Rod-like Molecules

**Figure 5:**
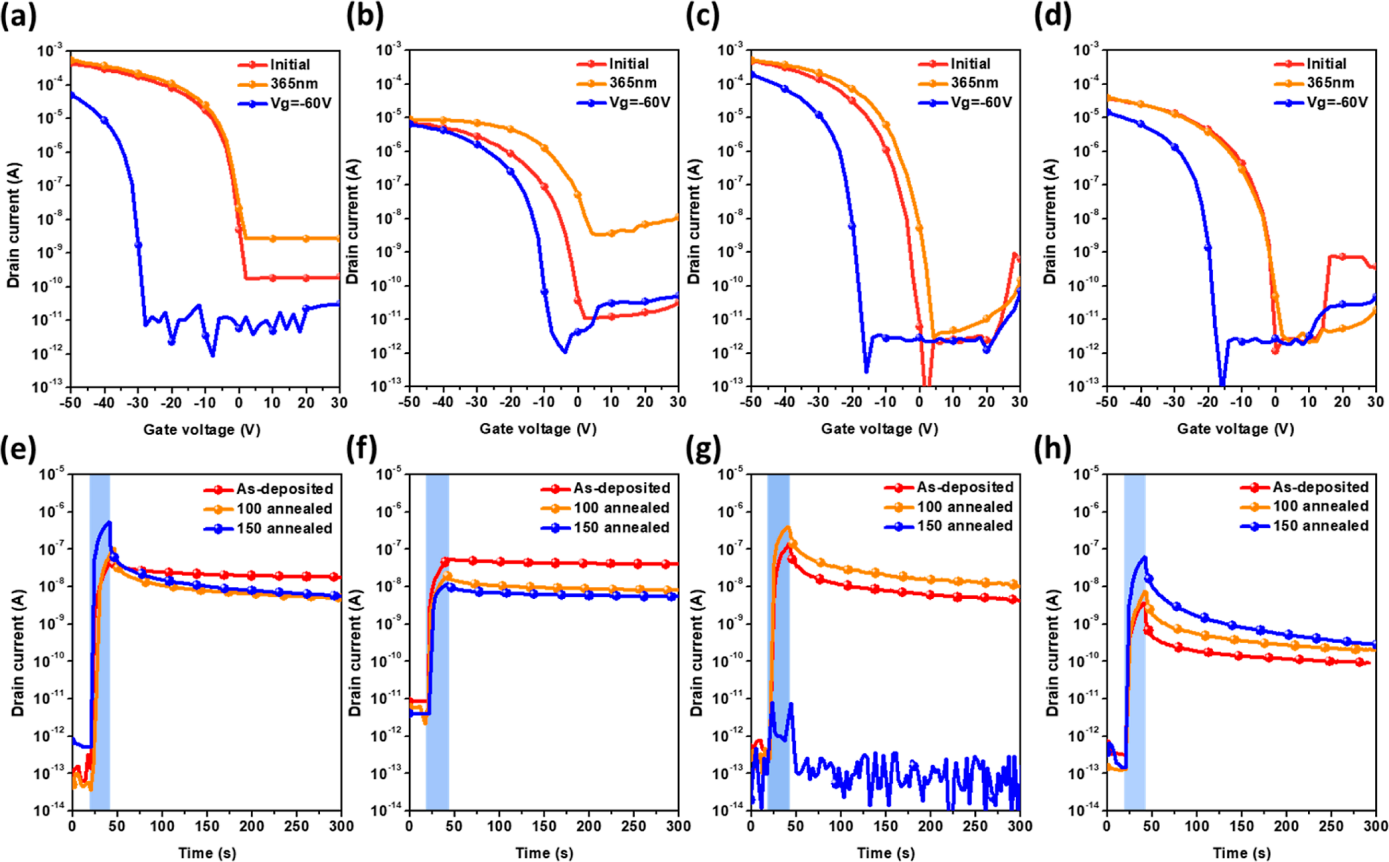
(a–d) Transfer characteristics and (e–f) transient characteristics of the devices comprising (a,e) C10-DNTT, (b,d) C10-6T, (c,g) C10-BTBT, and (d,h) C10-DNT. The transfer curves were measured under *V*_d_ = −50 V and *V*_g_ swept from 30 to −50 V. The photowriting or electrical erasing was conducted by illuminating 365 nm light (18 mW cm–2) for 20 s at *V*_d_ = −50 V or by applying a bias of *V*_g_ = −60 V for 5 s.

The OFET devices were prepared to comprehend the impact of liquid crystal structures on the electrical properties, carrier transport, and memory behavior. Furthermore, the devices were annealed at 100 and 150 °C after thermal evaporation to explore the influence of liquid crystal alignment on device performance. Note that C10-BTBT, C10-6T, and C10-DNTT were homeotropically aligned from the SmA, SmX, and SmX′ phases, respectively. The measurement was conducted at room temperature, so the rod-like molecular films were in polycrystalline states with smectic phase-aligned structures. The devices with the as-deposited and thermally annealed polycrystalline films were evaluated and compared. Figure 5a–d depicts the transfer curves; all these devices exhibited typical p-type hole-transport characteristics. The output curve of OFET based on C10-BTBT exhibited a typical p-type operation, as shown in Figure S13 (Supporting Information). Figure S14 displays the transfer curves of the films annealed at different temperatures. In the transfer characteristics, the gate voltage (*V*_g_) swept from 30 to −50 V, while the drain voltage (*V*_d_) was set to −50 V. As shown in Table 1, the average mobilities were measured as 0.70, 0.03, 0.87, and 0.06 cm^2^ V^–1^ s^–1^ for C10-DNTT, C10-6T, C10-BTBT, and C10-DNT, respectively. This disparity is related to the nature of the conjugated structure in the rod-like molecules. After annealing, it is observed that using the SmX-aligned structure for alignment results in similar trends of mobilities for C10-DNTT^43^ and C10-6T^40^ at 100 °C, which slightly decreases at 150 °C. This decrease might be due to the disordering of the molecular structure at higher temperatures. For C10-BTBT and C10-DNT, their mobilities slightly increase at 100 °C. At 150 °C, the mobility of the former decreases as it surpasses its melting point, while the latter, not exceeding the melting point, continues to increase.

**Table 1:** Summary of the Device Parameters, Including the Hole Mobility, Threshold Voltage, and Memory Window/Ratios, Measured from the Photonic Transistor Memory Comprising Rod-like Molecules in the As-deposited or Thermally Annealed States

	*T*_annealed_ [°C]^a^	μ_avg_ [c^2^ V^–1^ s^–1^]^b^	*V*_th,write_ [V]	*V*_th,erase_ [V]	*ΔV*_th_ [V]^c^	*I*_ON_/*I*_OFF_^d^	*I*_ON_/*I*_OFF_^e^
C10-DNTT	as-deposited	0.70 (1.01)	0.6	–8.9	9.5	5.3 × 106	6.3 × 10^3^
	100	0.92 (1.03)	–1.0	–11.1	10.1	1.1 × 107	
	150	0.54 (0.71)	0.4	–17.6	18.0	4.7 × 107	
C10-6T	as-deposited	0.03 (0.04)	2.2	–12.2	14.4	2.5 × 103	7.4 × 10^1^
	100	0.05 (0.07)	0.1	–21.8	21.8	4.7 × 103	
	150	0.02 (0.03)	0.0	–27.8	27.8	1.3 × 104	
C10-BTBT	as-deposited	0.87 (1.28)	–4.7	–21.9	17.3	4.3 × 105	1.7 × 10^3^
	100	2.32 (2.86)	–1.2	–22.2	21.0	1.5 × 10^6^	
C10-DNT	as-deposited	0.06 (0.08)	–4.5	–10.9	6.4	2.6 × 10^4^	N/A
	100	0.79 (0.96)	–2.4	–11.4	9.0	1.9 × 10^4^	
	150	0.50 (0.61)	–2.2	–13.2	11.0	4.6 × 10^5^	

a Post-treatment of the vacuum deposited thin films with different thermal annealing temperatures.

b Electron mobility derived from the saturated regime of the initial transfer characteristics. Note that the mobility is averaged from 3 different batches among 12 devices, and the values in parentheses are the measured maximum mobility among the devices.

c Memory window derived by the difference between the ON-state and OFF-state threshold voltage.

d Memory ratio derived from the current contrast of transfer curves in the ON/OFF states at *V*_g_ of 0 V.

e Memory ratio retained after 10,000 s at *V*_g_ = 0 V and *V*_d_ = −50 V.

To understand their memory behavior, external stimuli of light/bias were applied to conduct memory operations. The photowriting was conducted using 365 nm ultraviolet light. It was observed that under *V*_d_ = −50 V, the threshold voltage (*V*_th_) positively shifted to 0.6, 2.2, −4.7, and −4.5 V for C10-DNTT, C10-6T, C10-BTBT, and C10-DNT, respectively; when applying *V*_g_ = −60 V for 5 s, their *V*_th_ negatively shifted to −8.9, −12.2, −21.9, and −10.9 V. The memory window (*ΔV*_th_), defined as the difference between these two *V*_th_ are 9.5, 14.4, 17.3, and 6.4 for C10-DNTT, C10-6T, C10-BTBT, and C10-DNT, respectively. It can be observed that the first three devices have a larger *ΔV*_th_ compared to C10-DNT, indicating that they exhibit higher stability under photowriting operations. Correspondingly, in terms of memory ratio (*I*_ON_/*I*_OFF_), C10-BTBT has the highest value, followed by C10-DNTT and C10-6T, while C10-DNT shows a lower *I*_ON_/*I*_OFF_ comparatively. The photoassisted electrical writing was conducted by providing a positive *V*_g_ during photowriting, and the transfer curves and corresponding performance of 100 °C annealed C10-BTBT are, respectively, shown in Figure S15 and Table S4. This operation can further enhance the memory window by facilitating a deeper charge injection, thereby increasing the memory window. Regarding the impact of annealing temperature on device performance, Figure S16 demonstrates the devices’ transient characteristics pretreated by even higher annealing temperatures. Due to the deviation from the optimal homeotropic alignment temperature, this process leads to degraded device performance. Figure S17 shows the memory retention tests of the devices under 100 or 150 °C. The device performance has significantly decreased, and even applying a simultaneous electric field at 150 °C can result in device failure, resulting in an unmeasurable situation. As a result, the thermal energy has significantly reduced the effectiveness of the devices due to deteriorated charge trapping.

To understand their photoresponsivity, we measured their transient photoresponse. Figure 5e–h shows their temporal drain current curves. The photoswitching ratio was calculated by dividing the maximum photocurrent by the dark current, and photoresponsivity was calculated by dividing the maximum photocurrent by the light power in the active area. The photoswitching ratios are 1.1 × 10^6^, 8.9 × 10^3^, 1.7 × 10^6^, and 4.4 × 10^5^ for C10-DNTT, C10-6T, C10-BTBT, and C10-DNT, and the photoresponsivity values are 0.06, 0.01, 0.05, and 0.01 A W^–1^ for C10-DNTT, C10-6T, C10-BTBT, and C10-DNT, respectively. Note that the photoswitching ratio and photoresponsivity were from 150 °C-annealed C10-DNTT, 100 °C-annealed C10-6T, 100 °C-annealed C10-BTBT, and 150 °C-annealed C10-DNT. It can be observed that C10-DNTT exhibits the highest photocurrent, yielding the best photoresponsivity, while C10-BTBT has the highest photoswitching ratio due to the lowest dark current. It can be observed that C10-DNTT and C10-6T, which are arranged in the SmX-aligned phase,^40,43^ experience a decrease in photoresponse after annealing, but they exhibit relatively good overall current stability. In contrast, C10-BTBT, arranged in the SmA-aligned phase,^44^ shows an enhancement in photoresponse after annealing. C10-BTBT demonstrates a higher photoresponse than its SmX-aligned analogues of C10-DNTT and C10-6T. However, the photocurrent stabilities of C10-BTBT and C10-DNT are relatively poorer. This phenomenon can be attributed to the fact that C10-BTBT has a more ordered structure in the SmA-aligned structure, resulting in better charge-transport capabilities. After annealing, their overall regularity increases, reducing structural defects and limiting the ability for conformational changes, thereby reducing the available defects in the polycrystalline films. The well-arranged SmA-aligned structure restricts conformational variations induced by electric fields or light, giving them a higher charge-transport capability and photoresponse. There are fewer structural defects in a more ordered crystalline structure, such as C10-BTBT. Consequently, the inherent conformational changes induced by an electric field or light are relatively limited, leading to poorer current stability. In comparison, C10-DNTT and C10-6T with SmX-aligned structures provide good memory stability because of their relatively disordered structure, forming defects due to their inherent irregularity. This irregularity results in conformational variations under an electric field or light. Additionally, to obtain the memory switching speed, we set the target drain current to be 1 nA and calculated the illumination times required to achieve this target, which are 0.5, 0.5, 0.7, and 0.6 s, for 150 °C thermally annealed C10-DNTT, 100 °C thermally annealed C10-6T, 100 °C thermally annealed C10-BTBT, and 150 °C thermally annealed C10-DNT, respectively (Figure S18). The switching speed of the devices exhibits no significant deviation, which is presumably due to the comparable exciton dynamics of the LC molecules. Figure S19 shows a comparison of transient characteristics with different wavelengths of light. By comparing the UV absorption spectra in Figure S3, we observe that C10-DNTT and C10-DNT exhibit relatively higher absorbance at 310 nm than that of 365 nm light. Under the same irradiance of 49 μW cm^–2^, the devices’ photocurrent by 310 nm light irradiation is also higher compared to that of 365 nm light. On the other hand, with relatively higher absorbance for 365 nm light compared to 310 nm, C10-6T and C10-BTBT show higher photocurrent under the same irradiance of 49 μW cm^–2^. The result indicates a positive correlation between the absorbance and photoresponse.

**Figure 6:**
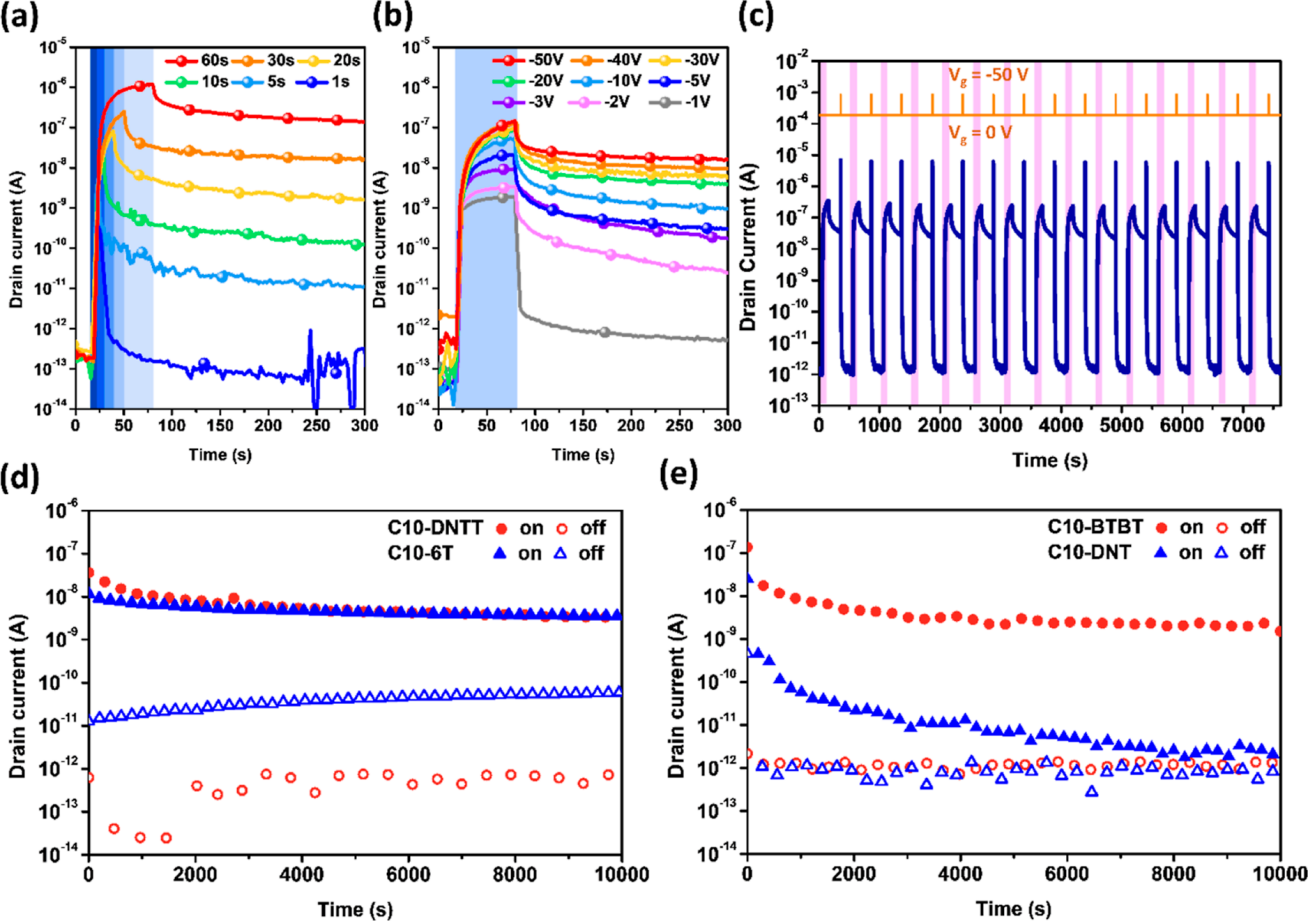
Photonic transistor memory characterization of C10-BTBT with different (a) photowriting durations and (b) drain voltage. (c) WRER cycles and memory retention tests of the photonic transistor memory comprising (d) C10-DNTT/C10-6T and (e) C10-BTBT/C10-DNT. The tests were conducted at *V*_d_ = −50 V during reading, photowriting (365 nm; 18 mW cm–2; 20 s), and electrical erasing (*V*_g_ = −60 V; 5 s).

Next, the impact of operational parameters on memory behavior was investigated. Using C10-BTBT as an example, the influence of light exposure duration (Figure 6a) and operating *V*_d_ (Figure 6b) on memory performance was characterized. Due to its well-organized smectic liquid crystal (SmA) arrangement, C10-BTBT demonstrated an excellent photoresponse. Under 5 s of light exposure or at −3 V, it achieved a high *I*_ON_/*I*_OFF_ of 3 orders of magnitude. This sufficiently represents its feasibility under low voltage and short light exposure times. Next, WRER (write–read–ease–read) operations were performed, where “W” stands for the photowriting process with 365 nm light at *V*_d_ = −50 V for 20 s, “E” represents the electrical erasing process at *V*_g_ = −60 V for 5 s, and “R” is the reading process under *V*_d_ = −50 V. In the WRER test of C10-BTBT, stable memory endurance was observed even after 15 consecutive cycles. Finally, to understand the memory stability, Figure 6c presents the stability analysis of the memory under *V*_d_ = −50 V for 10,000 s. It was observed that, except for C10-DNTT and C10-BTBT, which maintained approximately a 4-order *I*_ON_/*I*_OFF_, C10-6T showed nearly a 2-order *I*_ON_/*I*_OFF_. C10-DNT, on the other hand, did not exhibit distinguishable differences in memory levels. Notably, C10-DNTT, organized in an SmX-aligned arrangement, demonstrated the best memory stability. Conversely, C10-BTBT, arranged in an SmA-aligned arrangement, exhibited the best photoresponse; however, its stability was not as good as that of C10-DNTT.

### Working Mechanism of the Memory Behaviors in Rod-like Molecules

**Figure 7:**
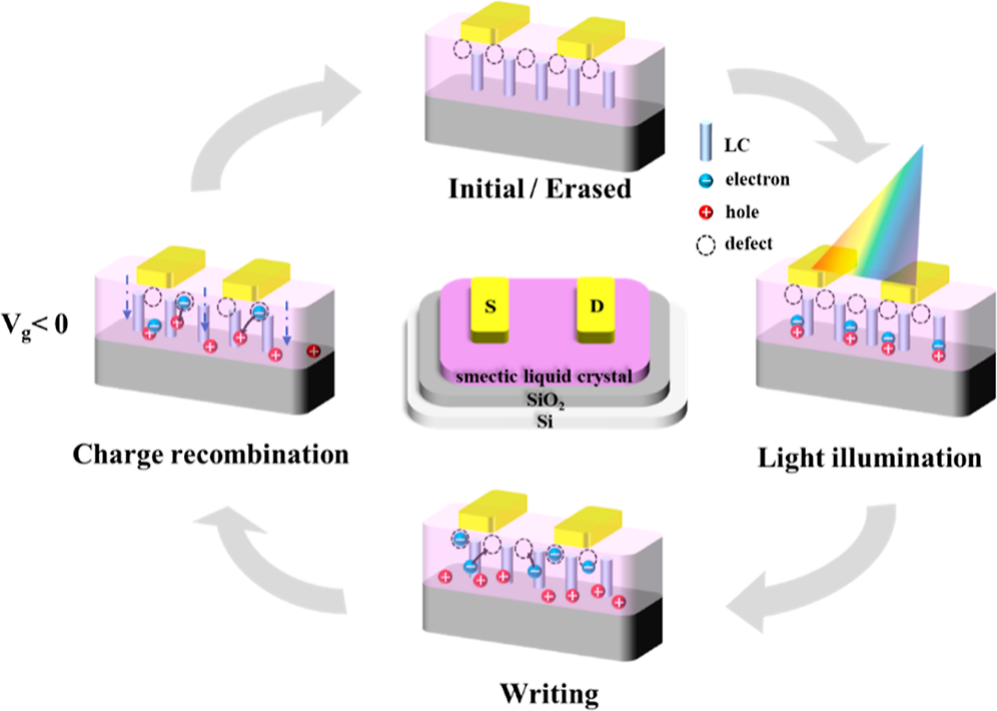
Schematic illustration of the photowriting and electrical erasing processes of the photonic transistor memory device conferred by the reversible molecular conformation transitions of smectic liquid crystals. Note that the polycrystalline region proximal to the SiO_2_ dielectric is defined as the hole channel of the device, and outside of this region are the underlying electron traps induced by the morphological inhomogeneity and reversible molecular conformation transitions.

To understand the relationship between the molecular structure, liquid crystal behavior, and memory performance of these rod-like molecules in photonic transistor memory, Figure 7 illustrates the operational schematic. When exposed to light, excitons are generated, leading to the storage of electrons and the extraction of holes, causing an increase in drain current. Accordingly, the device is switched to an ON state. When a negative bias voltage is applied, holes are injected from the external circuit of the device and neutralize the trapped electrons in the active layer. Consequently, recombination restores the device to the OFF state. This mechanism enables the switching of the ON/OFF states. In these devices, the rod-like molecules belong to the hole-transport channels, indicating that electron storage likely arises from morphology defects within the active layer and conformational variations. It is worth noting that the device measurement was conducted at room temperature, so the annealed films were in polycrystalline states with smectic phase-aligned structures. The GIXD results demonstrated that molecular conformation change was taking place by applying external stimuli of heat and bias. After investigating the memory devices systematically, the results demonstrated that memory behavior occurs in both the as-deposited (nanosprouts) and annealed (molecular flat) conditions. Therefore, in comparison to the structural inhomogeneity in the polycrystalline films, the light/bias-gated memory behavior is more likely to be contributed by reversible molecular conformation transitions induced by external stimuli.

Regarding the structures of these rod-like molecules, in a more ordered crystalline structure like C10-BTBT, there are fewer structural defects, and the inherent conformational changes induced by an electric field or light are relatively limited, leading to poorer current stability. In comparison, C10-DNTT and C10-6T provide good memory stability because of their rather disordered structure, forming defects due to their inherent irregularity and availability to conformational changes. The disorder induced by SmX-aligned structures in conformational variation and a higher number of morphology defects enrich it with charge traps. This leads to better memory stability. On the other hand, the homeotropic alignment conferred by the SmA phase has higher regularity, resulting in lower memory stability. However, they possess a more regular structure, enhancing their charge-transport capability. Consequently, the photoresponse and carrier mobility of C10-BTBT are superior and improved after annealing.

## Conclusions

In summary, a series of smectic liquid crystals were applied in photonic transistor memory under different conditions to clarify the light-bias-gating mechanism and origin of memory behavior. The side-chain length of these rod-like molecules was carefully selected to control the phase transition region of smectic liquid crystals. The homeotropically aligned smectic liquid crystals served as both hole-transport channels and electron traps in the devices. Attributed to the 3D-ordered structure of the smectic liquid crystals, the memory device exhibited a good response with photowriting and electrical erasing processes. The photomemory device of C10-BTBT, due to its SmA-aligned structure, shows a relatively higher mobility than other structures. After annealing, causing the enhancement of homeotropic alignment, its *I*_ON_/*I*_OFF_ can even exceed 10^6^. After investigating the memory devices systematically, the results demonstrated that memory behavior occurs under both the as-deposited and annealed conditions, implying a more predominant contribution from reversible molecular conformation transitions than structural inhomogeneity in the polycrystalline films. In summary, the rod-like molecules exhibit high potential for developing nonvolatile memory with a combined single-layered structure of semiconducting/memory layers. These findings help us better understand the application of smectic liquid crystals in photomemory devices.

## Supporting Information Available

Device characteristics of the photomemory; water contact angles of the polycrystalline molecule films; electrochemical and optical characterization of the constituent materials; crystallographic parameters of the rod-like molecule films; 1D height profiles of the films derived from the AFM topographies; and output characteristics of the OFET device (PDF)
