# Supplementary material for: Reversible Molecular Conformation Transitions of Smectic Liquid Crystals for Light/Bias-Gated Transistor Memory
Source: ACS Appl Mater Interfaces. 2024 Feb 1;16(6):7500–11. doi: 10.1021/acsami.3c16882 (PMC10875644; doi:10.1021/acsami.3c16882)
Supplement: Supplementary file 1 — am3c16882_si_001.pdf [file am3c16882_si_001.pdf]

Supporting Information

Reversible Molecular Conformation Transitions of Smectic Liquid Crystals for Light/Bias-Gated Transistor Memory

Yi-Chieh Neu,[a] *Yi-Sa Lin,*[a] *Yi-Hsun Weng,*[a] *Wei-Cheng Chen,*[a] *Cheng-Liang Liu,*[b,c] *Bi-Hsuan Lin,*[d] *Yan-Cheng Lin,*[c,e*] *and Wen-Chang Chen*[a,c,*]

[a] Department of Chemical Engineering, National Taiwan University, Taipei 10617, Taiwan

[b] Department of Materials Science and Engineering, National Taiwan University, Taipei 10617, Taiwan

[c] Advanced Research Center for Green Materials Science and Technology, National Taiwan University, Taipei 10617, Taiwan

[d] National Synchrotron Radiation Research Center, Hsinchu 30076, Taiwan

[e] Department of Chemical Engineering, National Cheng Kung University, Tainan 70101, Taiwan

*Corresponding author. E-mail: ycl@gs.ncku.edu.tw (Y.-C. Lin); chenwc@ntu.edu.tw (W.-C. Chen)

Table S1. The parameters derived from TR-PL 1D decaying profiles of the thermally annealed rod-like molecule films. The averaged lifetime ($\tau_{average}$) is calculated by $\tau_{average} = A_1\tau_1^2/(A_1\tau_1 + A_2\tau_2) + A_2\tau_2^2/(A_1\tau_1 + A_2\tau_2)$.

	A_1	τ_1 (ns)	A_2	τ_2 (ns)	$\tau_{average}$ (ns)
C10-BTBT	0.557	0.352	0.557	0.352	0.352
C10-DNT	0.730	1.581	0.256	0.270	1.507
C10-DNTT	1.000	0.262	0.048	1.407	0.497

Table S2. The surface energies derived from the water contact angle (CA) of the as-deposited or thermally annealed rod-like molecule films. The calculations followed the Owens−Wendt contact angle model with the polar and dispersion contributions.

	$T_{annealing}$ (°C)	σ_s^P (mJ m^{-2})	σ_s^D (mJ m^{-2})	σ_s (mJ m^{-2})
C10-DNTT	As-deposited	0.11	16.88	16.99
	100	1.93	34.65	36.58
	150	0.04	24.83	24.87
C10-BTBT	As-deposited	0.12	23.75	23.87
	100	0.29	20.83	21.11
C10-DNT	As-deposited	5.99	6.03	12.02
	100	0.59	22.72	23.31
	150	0.59	19.42	20.01
C10-6T	As-deposited	1.27	16.25	17.52
	100	1.00	18.09	19.09
	150	1.00	16.67	17.67

Table S3. Crystallographic parameters of the as-deposited or thermally annealed rod-like molecule films, and the thermally annealed thin films under different treatments including heating at 100°C for C10-BTBT and C10-6T, and 150°C for C10-DNTT and C10-DNT or applying a vertical bias of 2 V.

	Condition	q_z* [nm^{-1}][a]	d_{001} [nm][a]	FWHM [nm^{-1}][b]	L_c [nm][b]
C10-DNTT	As-deposited	1.64	3.83	0.30	18.85
	Annealed	1.64	3.83	0.17	33.26
	Heating	1.64	3.83	0.20	28.27
	Electric Field	1.64	3.83	0.25	22.62
C10-BTBT	As-deposited	1.94	3.24	0.12	47.12
	Annealed	1.85	3.40	0.11	51.41
	Heating	1.94	3.24	0.10	56.55
	Electric Field	1.89	3.32	0.14	40.39
C10-DNT	As-deposited	1.77	3.55	0.17	33.26
	Annealed	1.76	3.57	0.11	51.41
	Heating	1.80	3.49	0.12	47.12
	Electric Field	1.76	3.57	0.19	29.76
C10-6T	As-deposited	1.52	4.13	0.28	20.20
	Annealed	1.52	4.13	0.27	20.94
	Heating	1.47	4.27	0.15	37.70
	Electric Field	1.43	4.39	0.97	5.83

[a] The *d*-spacing calculated by the highest peak of the out-of-plane (001) diffraction; [b] Full-width at half maximum of (001) diffraction; [c] Crystallite size calculated by Scherrer equation: $L_c = 0.9 \times 2\pi / \text{FWHM}$.

Table S4. Summary of the device performance under varied photo-assisted electrical writing states, including the hole mobility, threshold voltage, and memory window.

	V_d [V] [a]	V_g [V] [a]	μ_{avg} [cm^2 V^{-1} s^{-1}] [b]	$V_{th, write}$ [V]	$V_{th, erase}$ [V]	ΔV_{th} [V] [c]
C10-BTBT 100°C annealed	–50	0	0.24 (0.32)	–9.2	–22.2	13
	–40	10	0.18 (0.24)	–7.2	–21.9	14.7
	–30	20	0.18 (0.21)	–4.0	–22.0	18
	–20	30	0.24 (0.23)	–2.9	–22.0	19.1
	–10	40	0.21 (0.22)	–0.3	–22.3	22

[a] The drain/gate voltage applied during the photo-assisted electrical writing processes. [b] Hole mobility derived from the saturation regime of the initial transfer characteristics. Note that the mobility is averaged from 3 different batches, and the values in parenthesis are the measured maximum mobility among the devices. [c] Memory window derived by the difference between the ON-state and OFF-state threshold voltages.

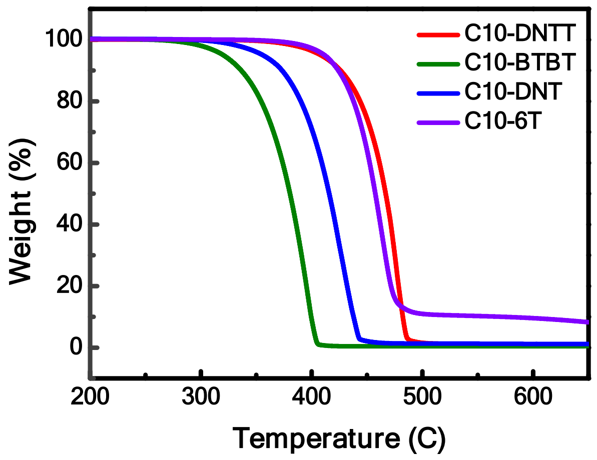

Figure S1. TGA profiles of the rod-like molecules, C10-DNTT, C10-BTBT, C10-DNT, and C10-6T. Note that the measurements were all ramped at 10 °C/min to 650 °C under a nitrogen atmosphere.

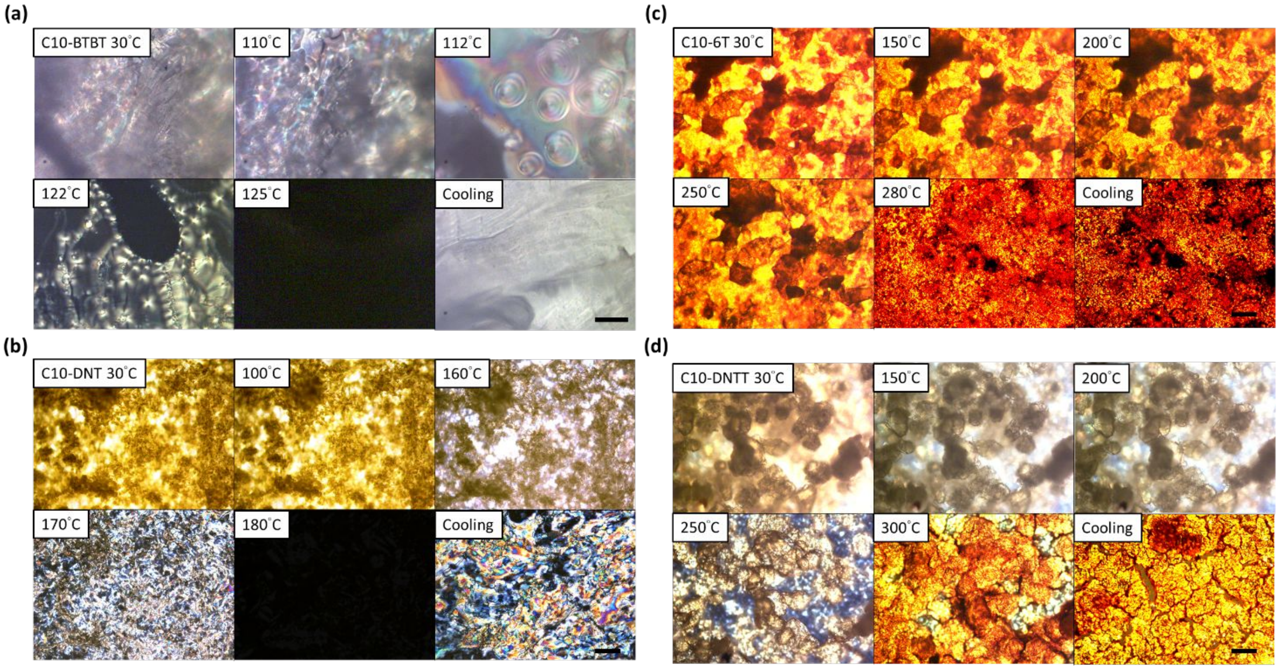

Figure S2. POM images of the drop-casted films of (a) C10-BTBT (b) C10-DNT (c) C10-6T (d) C10-DNTT at different temperatures. Note that the cooling state was captured at room temperature. The scale bars are (a) 25 μm, (b–d) 100 μm.

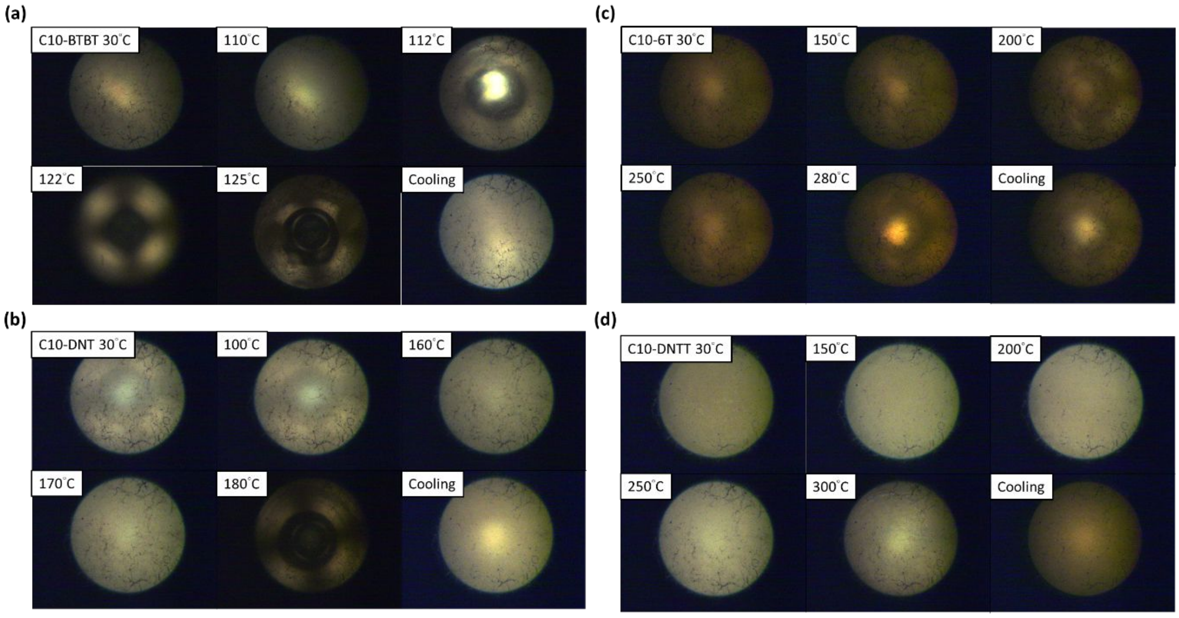

Figure S3. Conoscopies of the drop-casted films of (a) C10-BTBT (b) C10-DNT (c) C10-6T (d) C10-DNTT at different temperatures.

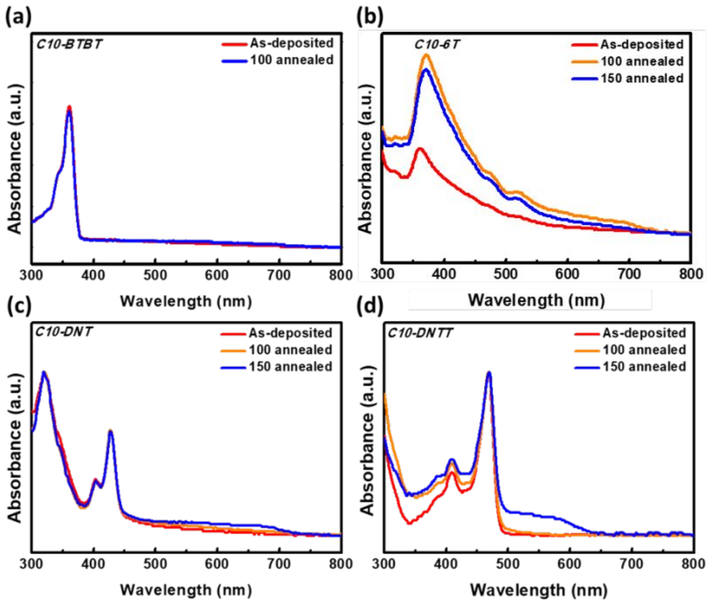

Figure S4. The optical absorbance spectra for (a) C10-BTBT (b) C10-6T (c) C10-DNT (d) C10-DNTT at different annealing temperatures.

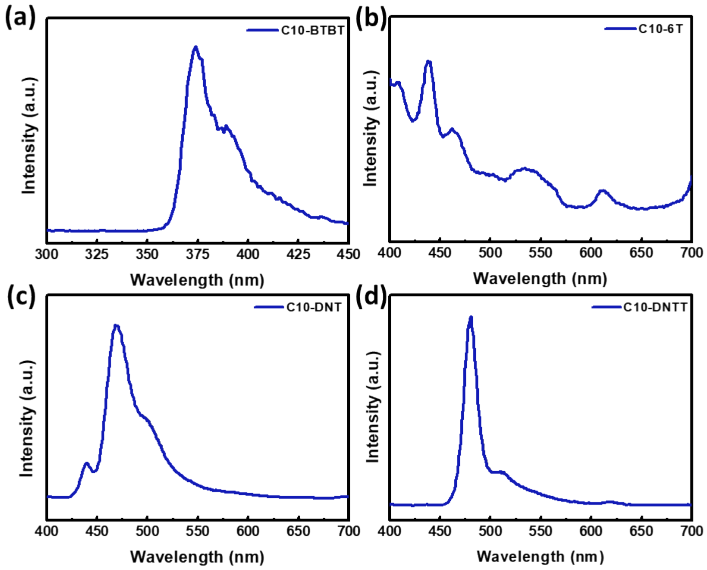

Figure S5. The PL emission spectra for (a) C10-BTBT (b) C10-6T (c) C10-DNT (d) C10-DNTT. Note that the excitation wavelengths were 350, 365, 320, and 365 nm for C10-BTBT, C10-6T, C10-DNT, and C10-DNTT, respectively.

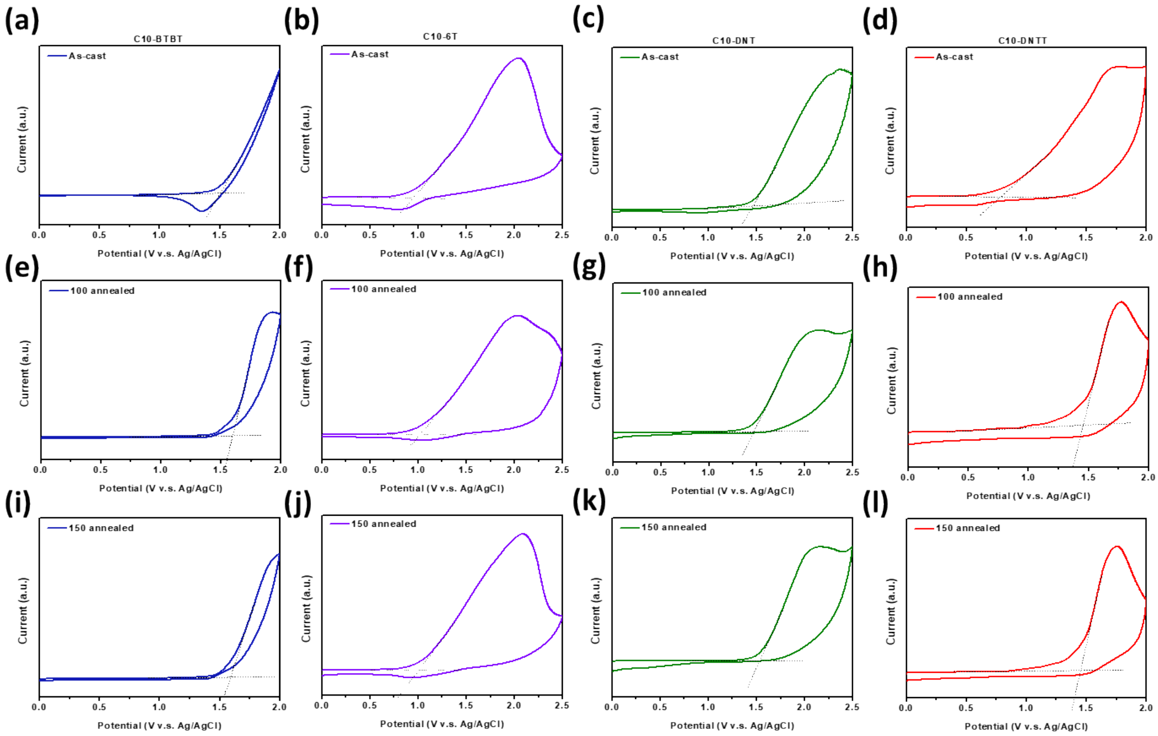

Figure S6. CV profiles of (a,e,i) C10-BTBT (b,f,j) C10-6T (c,g,k) C10-DNT (d,h,l) C10-DNTT thin films spin-coated onto the ITO glass (a–d) without annealing (e–h) annealed at 100 °C (i–l) annealed at 150 °C. Note that the measurement was conducted in a three-electrode system, in which Ag/AgCl and Pt rod were used as a reference and a counter electrode, respectively. The sweeping rate was fixed at 100 mV s^{-1}.

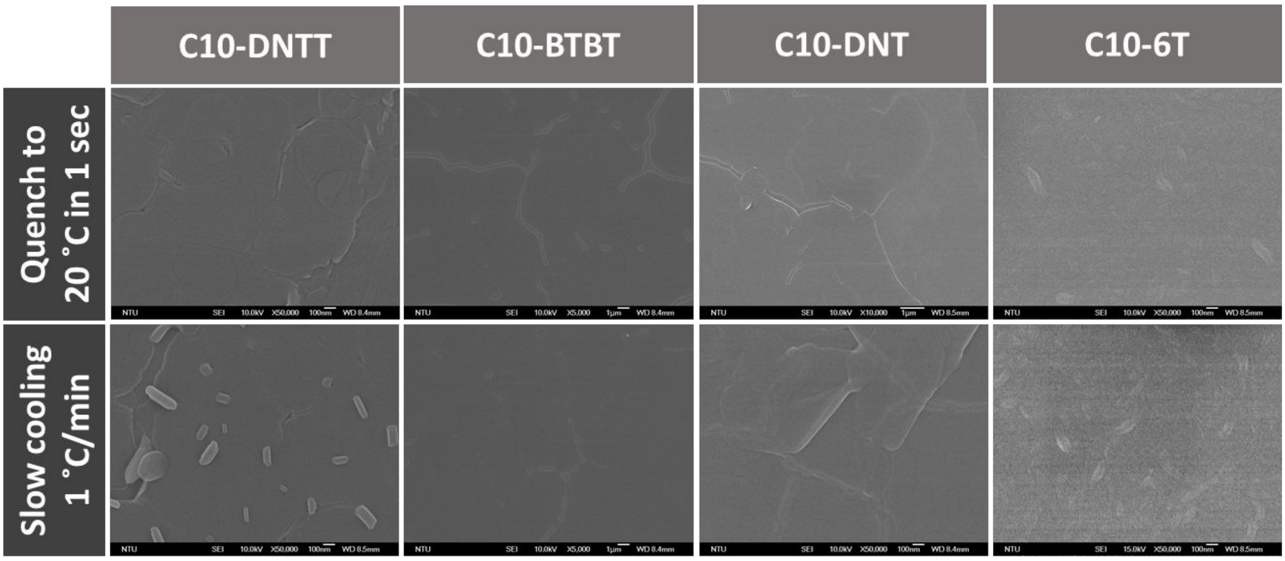

Figure S7. SEM images of samples with different cooling rates in the annealing process. The polycrystalline films were heated to 150 °C for C10-DNTT and C10-DNT, 100 °C for C10-BTBT, and C10-6T to promote the solid-state stacking by homeotropic alignment.

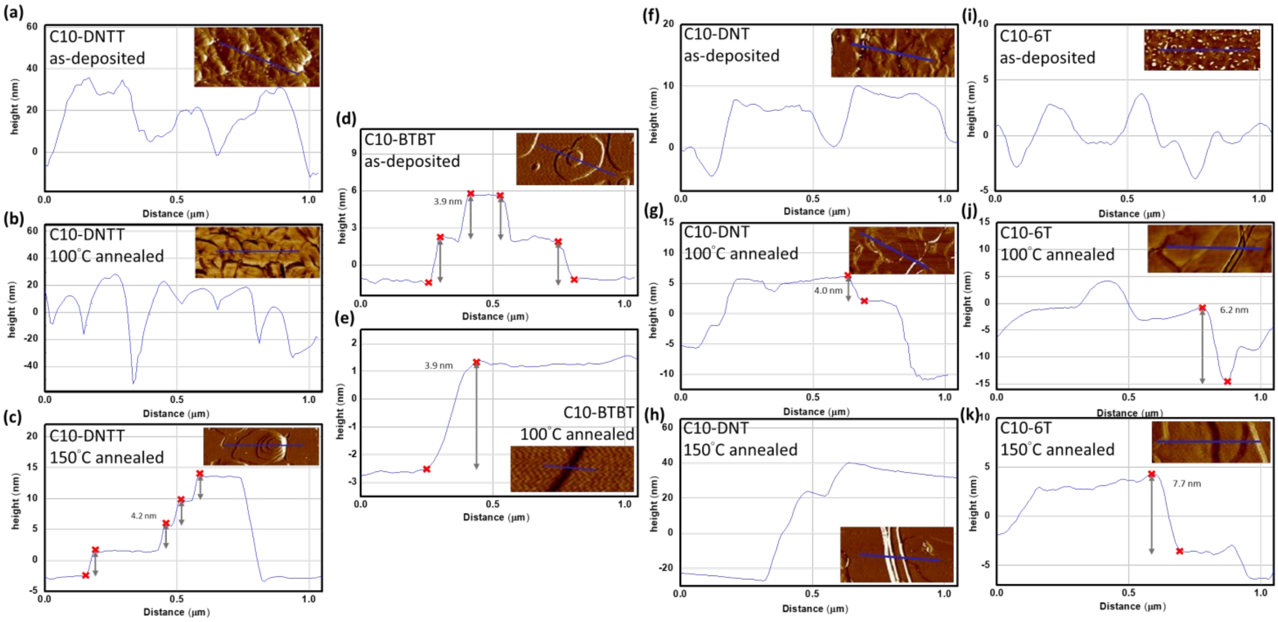

Figure S8. 1D height profiles derived from the AFM topographies of the (a–c) C10-DNTT (d,e) C10-BTBT (f–h) C10-DNT (i–k) C10-BTBT films in the as-deposited states (a,d,f,i) or after thermal annealing at different temperatures of (b,e,g,j) 100 °C or (c,h,k) 150 °C. The inset figures attached to the profiles show the corresponding 2D height images.

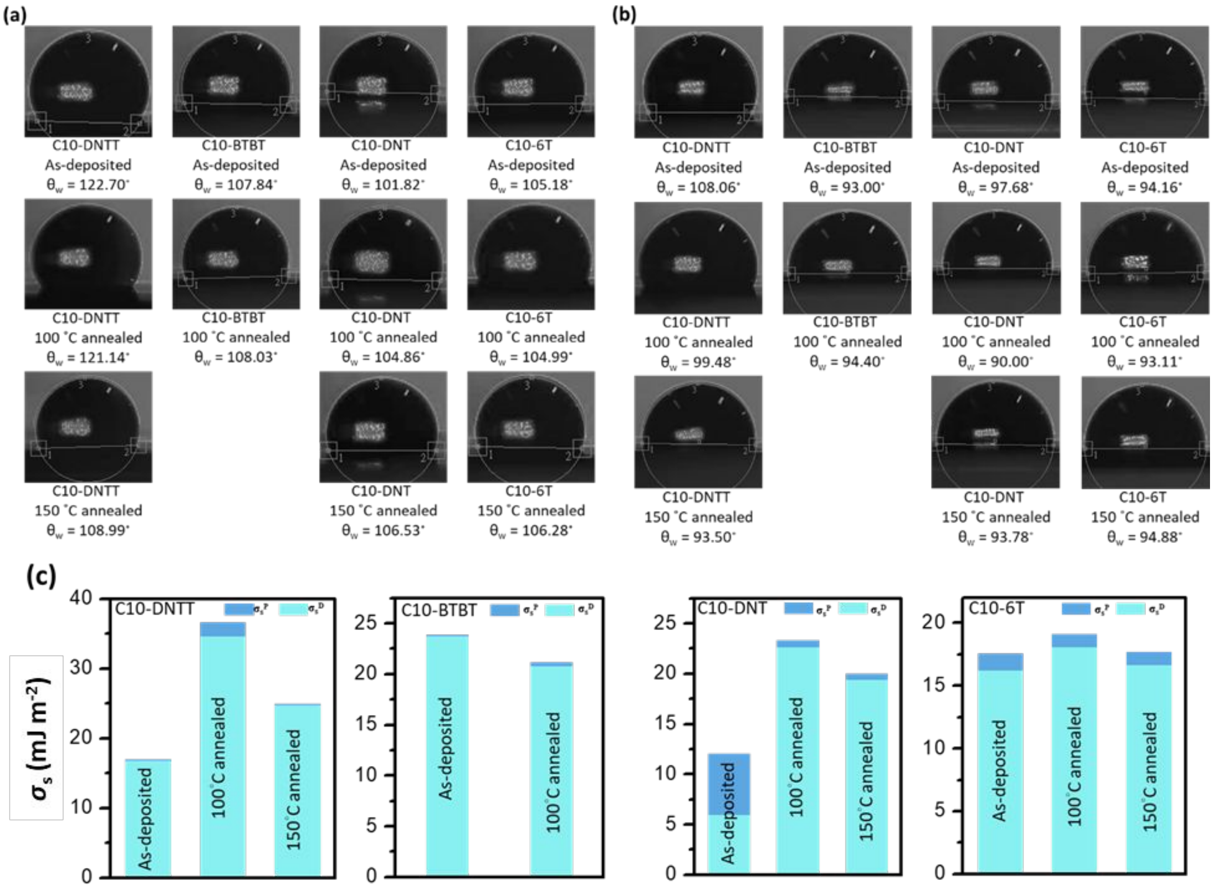

Figure S9. (a) Water and (b) glycerol contact angles of the thin films. (c) The corresponding surface energy bar charts based on the calculations of the contact angles with the Owens−Wendt contact angle model.

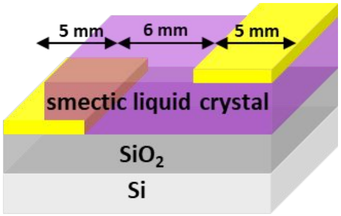

Figure S10. The device for the XRD measurement applying an out-of-plane direction electric field was fabricated based on a Si substrate with a 100-nm-thick SiO_2 layer. First, Au was deposited on the surface by thermal evaporation through a mask, forming a 50-nm-thick gold electrode. Next, the liquid crystal molecules were deposited on the top, while the thickness was 50 nm. Finally, 50 nm thick gold was deposited to form the other electrode. The gap between the bottom-layered and top-layered Au electrodes was 6 mm. These electrodes were connected to a controllable DC power supply to apply an electric field with a bias of 2 V.

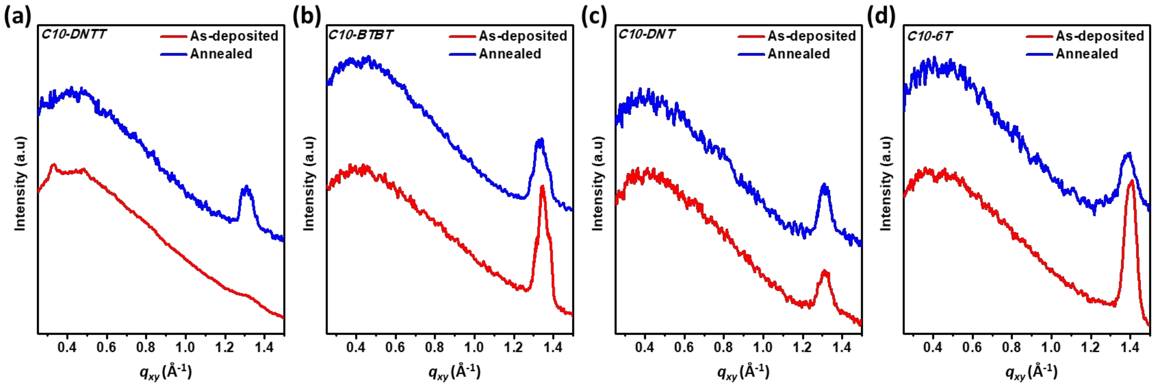

Figure S11. 1D GIXD line-cutting profiles of the (a) C10-DNTT, (b) C10-BTBT, (c) C10-DNT, and (d) C10-6T along the in-plane direction. Note that the measurements were based on the as-deposited or thermally annealed rod-like molecule films at 100°C for C10-BTBT and C10-6T and 150°C for C10-DNTT and C10-DNT.

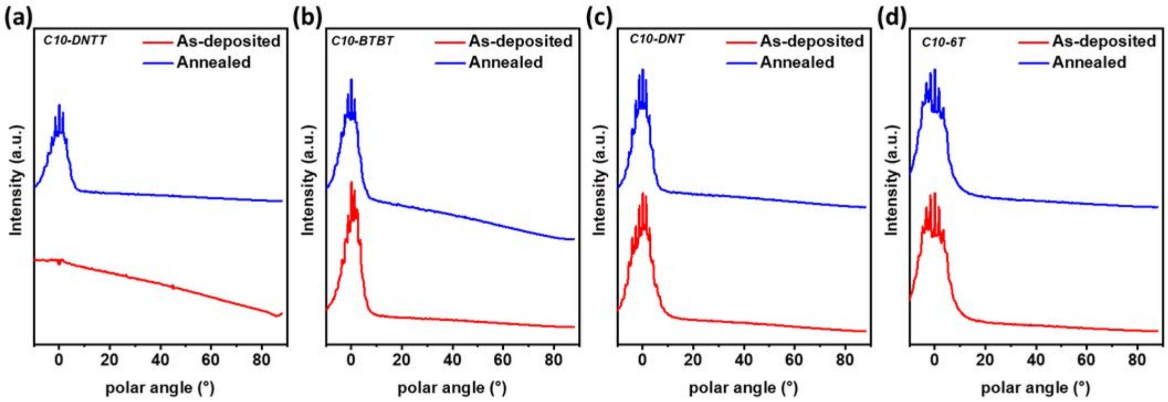

Figure S12. The geometrically corrected and normalized pole figures of (a) C10-DNTT, (b) C10-BTBT, (c) C10-DNT, and (d) C10-6T, with 0° and 90° represent the out-of-plane and in-plane directions. Note that the measurements were based on the as-deposited or thermally annealed rod-like molecule films at 100°C for C10-BTBT and C10-6T and 150°C for C10-DNTT and C10-DNT.

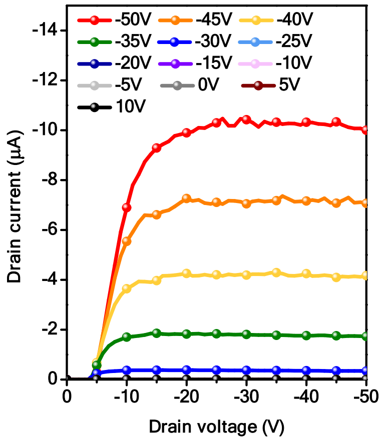

Figure S13. The output curves of the thermally annealed C10-BTBT at different gate voltages.

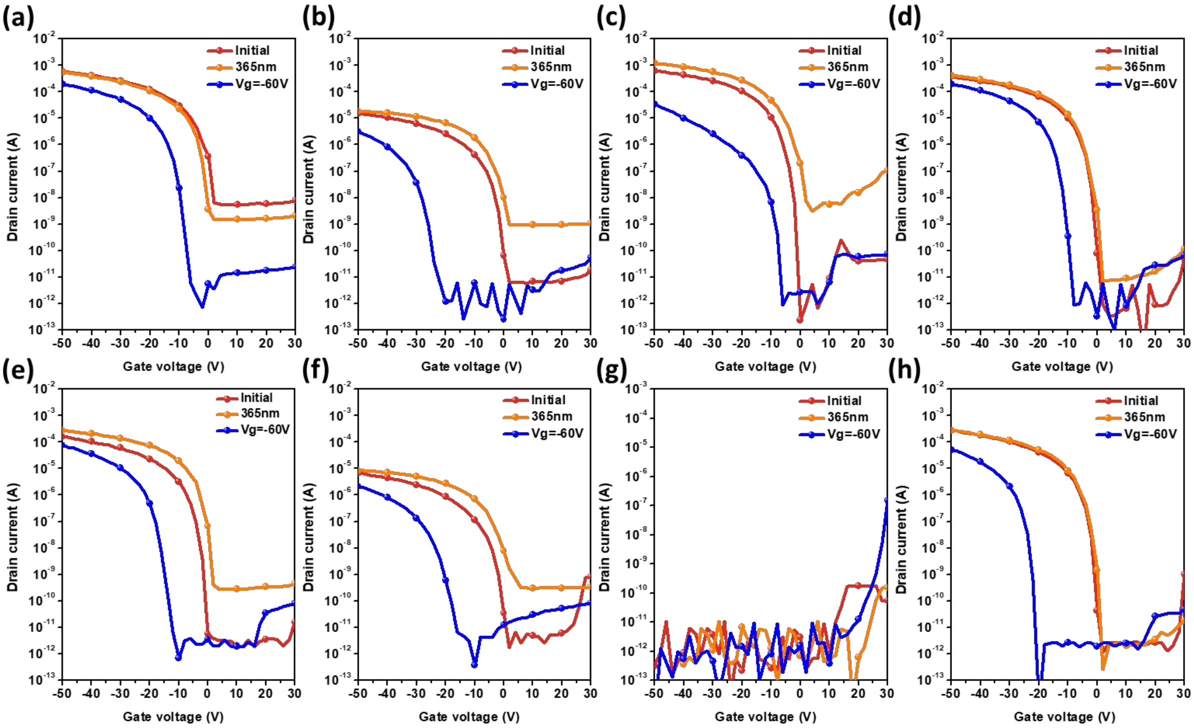

Figure S14. Transfer characteristics of the devices comprising (a,e) C10-DNTT, (b,d) C10-6T, (c,g) C10-BTBT, and (d,h) C10-DNT after annealing at (a−d) 100 °C or (e−f) 150 °C . Note that the transfer curves were measured under $V_d = -50$ V and V_g from 30 to −50 V, and the photo-writing or electrical erasing in both characteristics was conducted by applying 365-nm light for 20 s at $V_d = -50$ V or $V_g = -60$ V for 5 s.

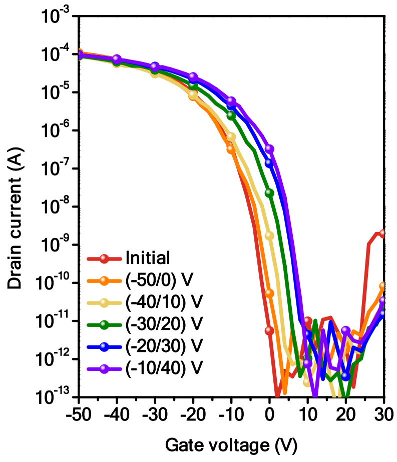

Figure S15. Transfer characteristics of the thermally annealed C10-BTBT at different (drain voltage/ gate voltage) during the photo-writing stage. Note that the transfer curves were measured under $V_d = -50$ V and V_g from 30 to −50 V and the photo-writing was conducted by applying 365-nm light for 20 s at $V_d = -50$ V.

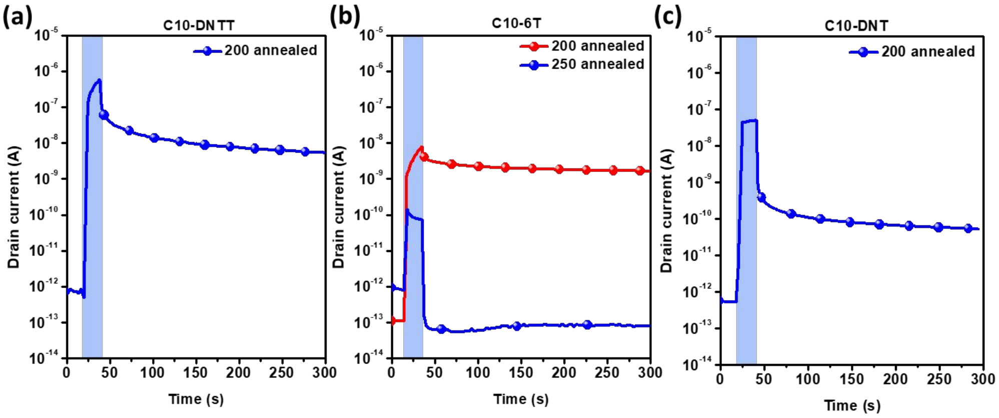

Figure S16. Transient characteristics of the devices comprising (a) C10-DNTT, (b) C10-6T, and (c) C10-DNT. Due to the deviating from the appropriate homotropic alignment temperature, resulting in a decline in device performance. Note that photo-writing was conducted by applying 365-nm light for 20 s at $V_{\mathrm{d}} = -50$ V.

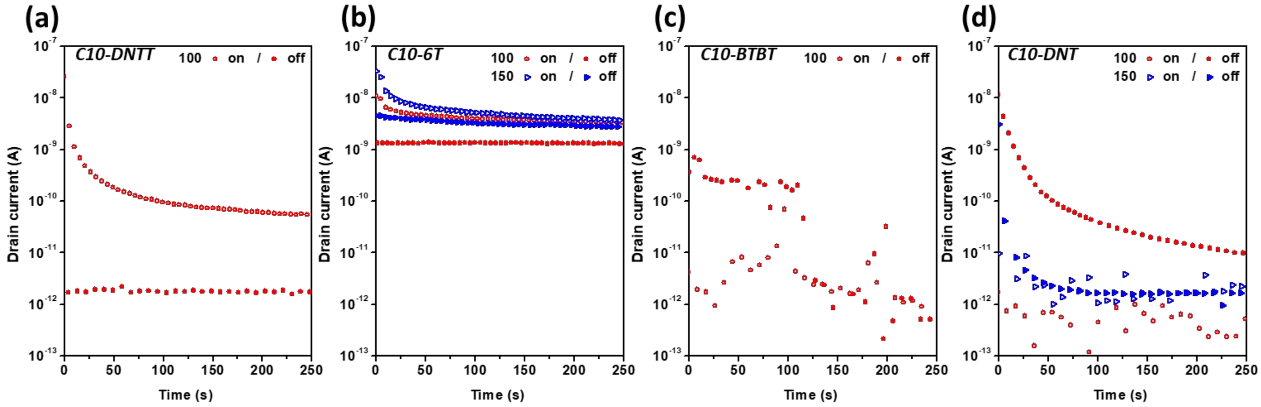

Figure S17. Memory retention tests of the (a) C10-DNTT, (b) C10-6T, (c) C10-BTBT, and (d) C10-DNT device under 100 or 150°C. The device performance has significantly decreased, and even applying an electric field at 150 °C simultaneously can result in device failure, rendering it unmeasurable. Note that the tests were conducted at $V_{\mathrm{d}}= -50$ V during reading, and photo-writing (365 nm; 18 mW cm^{-2}; 20 s)

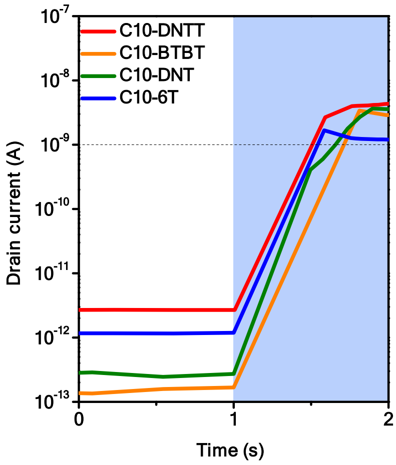

Figure S18. Memory switching speed tests for C10-DNTT, C10-BTBT, C10-DNT, and C10-6T. Note that the devices were thermally annealed at 100°C for C10-BTBT and C10-6T and 150°C for C10-DNTT and C10-DNT. And photo-writing was conducted by applying 365-nm (18 mW cm^{-2}) light at $V_d = -50$ V.

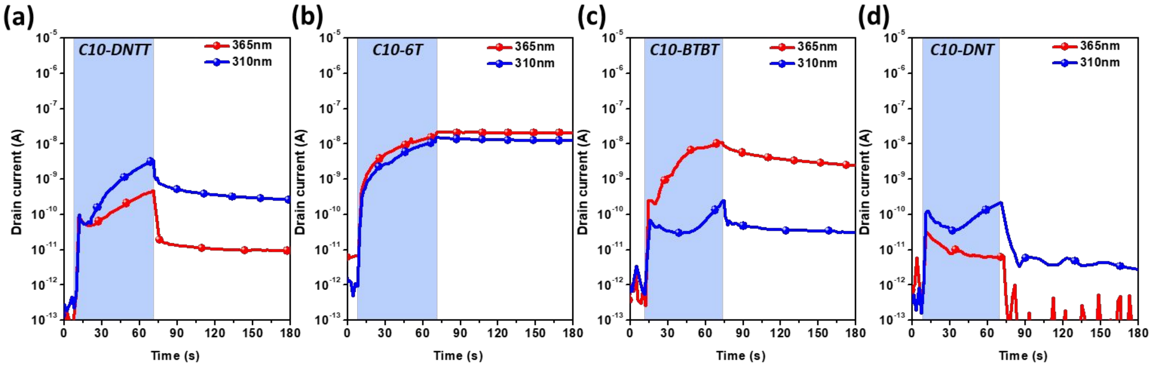

Figure S19. Transient characteristics of the devices comprising (a) C10-DNTT, (b) C10-6T, (c) C10-BTBT, and (d) C10-DNT without annealing. Note that photo-writing was conducted by applying 310-nm (49 µW cm^{-2}) and 365-nm (49 µW cm^{-2}) light for 60 s at $V_d = -50$ V.
